# Association of papillomavirus E6 proteins with either MAML1 or E6AP clusters E6 proteins by structure, function, and evolutionary relatedness

**DOI:** 10.1371/journal.ppat.1006781

**Published:** 2017-12-27

**Authors:** Nicole Brimer, Camille M. Drews, Scott B. Vande Pol

**Affiliations:** Department of Pathology, University of Virginia, Charlottesville, Virginia, United States of America; University of Wisconsin Madison School of Medicine and Public Health, UNITED STATES

## Abstract

Papillomavirus E6 proteins bind to LXXLL peptide motifs displayed on targeted cellular proteins. Alpha genus HPV E6 proteins associate with the cellular ubiquitin ligase E6AP (UBE3A), by binding to an LXXLL peptide (ELTLQELLGEE) displayed by E6AP, thereby stimulating E6AP ubiquitin ligase activity. Beta, Gamma, and Delta genera E6 proteins bind a similar LXXLL peptide (WMSDLDDLLGS) on the cellular transcriptional co-activator MAML1 and thereby repress Notch signaling. We expressed 45 different animal and human E6 proteins from diverse papillomavirus genera to ascertain the overall preference of E6 proteins for E6AP or MAML1. E6 proteins from all HPV genera except Alpha preferentially interacted with MAML1 over E6AP. Among animal papillomaviruses, E6 proteins from certain ungulate (SsPV1 from pigs) and cetacean (porpoises and dolphins) hosts functionally resembled Alpha genus HPV by binding and targeting the degradation of E6AP. Beta genus HPV E6 proteins functionally clustered with Delta, Pi, Tau, Gamma, Chi, Mu, Lambda, Iota, Dyokappa, Rho, and Dyolambda E6 proteins to bind and repress MAML1. None of the tested E6 proteins physically and functionally interacted with both MAML1 and E6AP, indicating an evolutionary split. Further, interaction of an E6 protein was insufficient to activate degradation of E6AP, indicating that E6 proteins that target E6AP co-evolved to separately acquire both binding and triggering of ubiquitin ligase activation. E6 proteins with similar biological function clustered together in phylogenetic trees and shared structural features. This suggests that the divergence of E6 proteins from either MAML1 or E6AP binding preference is a major event in papillomavirus evolution.

## Author summary

Papillomaviruses are a large family of viruses with great medical and veterinary importance. This study explores the viral E6 oncoproteins from diverse papillomavirus genera to determine how E6 distinguishes in interaction between cellular proteins. E6 proteins have been previously found to interact with a ubiquitin ligase called E6AP and thereby target particular cellular proteins for degradation, or to interact with MAML family proteins to repress Notch signaling and thereby alter cellular differentiation. It has been unclear if diverse families of papillomavirus E6 proteins interact with only E6AP or MAML (or possibly both), how E6 distinguishes between these interactions, and if interaction of E6 with E6AP is coupled to ubiquitin ligase activation. We find here that none of the tested E6 proteins physically and functionally interacted with both E6AP and MAML1, indicating an evolutionary split that clustered E6 proteins by sequence similarity analysis. Currently, the categorization of papillomaviruses is complex, with thirty-eight genera so far described. This study establishes an early evolutionary split among most papillomavirus genera between those viruses that encode E6 proteins that physically and functionally associate with MAML compared to E6AP. This provides a structural and functional basis for categorizing most currently described papillomaviruses into two major functional groups.

## Introduction

Papillomaviruses are a large group of viruses with hundreds of different fully sequenced types and additional types partially characterized by metagenomic sequencing [1–4]. All papillomaviruses express early genes E1 and E2 that are necessary for viral transcriptional control and DNA replication, as well as late gene capsid proteins L1 and L2 that package progeny viral DNA. Almost all papillomaviruses also express accessory early proteins with oncogenic properties, which are subject to transcriptional control by E1 and E2 (termed E5, E6, E7, and other designations) [5–9]. While many papillomaviruses encode all three oncoproteins, one or more of the oncoproteins may be absent within a particular group of related papillomaviruses. Papillomaviruses are classified based upon nucleotide sequence similarity of the major L1 capsid protein into taxonomic levels of genus (different genera share less than 60% nucleotide sequence identity in L1), species (share between 60% and 70% nucleotide identity), and types (types within a species are between 71% and 89% identical in sequence within L1) [10]. Thirty-eight genera of papillomaviruses have been described, with additional genera expected as additional animal papillomaviruses will be detected in the future [4, 11].

Papillomavirus clustering on the basis of early gene relatedness instead of L1 has also been performed [12, 13], and shows more coherent clustering of E6 by clinical phenotype compared to L1 [14]. However, L1 clustering is practical, primarily because L1 is more highly conserved than the early region genes, and all papillomaviruses encode the L1 gene. But given the large and growing number of genera, a parallel approach that clusters papillomaviruses into smaller numbers of groups with biological relatedness might be more comprehensible.

Although all virus-induced papillomas are initially benign, some virus types produce papillomas that may progress into malignancy. Some Alpha genus human papillomaviruses (HPVs) are associated with anogenital and upper airway cancers in primates (reviewed in [15]), and certain animal papillomaviruses (such as cotton tailed rabbit papillomavirus [16], and Ovies Aires Papillomavirus type 3 [17], produce cutaneous papillomas that can progress to malignancy. The subset of HPVs associated with cancer is referred to as “high-risk” HPV types (HPV types 16, 18, and 31 are model systems), and the related mucosal Alpha genus viruses that do not cause malignancies are called “low-risk” HPVs (prototypes being HPV types 6 and 11) [18].

The propensity of a particular papillomavirus to produce a cancer is a property of the virally encoded oncoproteins, and in some viruses, environmental exposure to carcinogens (such as Bovine Papillomavirus type 2 when cows consume bracken fern [19]). High-risk HPVs encode an E7 oncoprotein that associates with retinoblastoma family proteins and targets them for degradation, thereby ablating cell cycle checkpoint control and contributing to genomic instability [20]. The HPV high-risk E6 oncoproteins associate with a cellular E3 ubiquitin ligase called E6AP (a product of the UBE3A gene) [21]. E6 associates with E6AP by docking upon an alpha-helical LXXLL-containing peptide motif in E6AP [21–24]. Upon binding an LXXLL peptide, E6 undergoes a conformational change that promotes the association of E6 with p53 [25], p53 ubiquitination by E6AP, and p53 degradation by the proteasome. Therefore, like E7, high-risk E6 promotes genomic instability [26]. Low-risk mucosal HPV types also express E7 proteins but these do not target the degradation of Retinoblastoma (RB) [20]. While low-risk E6 proteins bind to E6AP and trigger E6AP degradation, they have not been shown to interact with p53 [27].

It is clear that E6 proteins from non-Alpha genera do not primarily associate with E6AP. The Delta genus E6 protein from Bovine Papillomavirus type 1 (BPV1 E6) associates with focal adhesion proteins paxillin (PXN) and HIC5 by binding to LXXLL motifs on those proteins that are similar to but distinct from the LXXLL motif of E6AP [28, 29]. PXN expression, BPV1 E6 interaction with LXXLL motifs, and docking of BPV1 E6 on particular LXXLL motifs of PXN are all required for BPV1 E6 to induce anchorage-independent colony formation [30–33]. Recently, E6 proteins from HPV Beta genus cutaneous papillomaviruses as well as BPV1 E6 were found to interact with MAML1 transcriptional coactivators, and thereby repress Notch signaling [34–37]. The interaction of these E6 proteins with MAML1 resembled that of BPV1 E6 with PXN and HIC5, and HPV16 E6 protein with E6AP, in that the cutaneous type E6 proteins bound to an LXXLL peptide motif located at the carboxy-terminus of MAML1. BPV1 E6 and Beta genus HPV E6 proteins preferentially interact with MAML1 compared to E6AP despite the similarity of the LXXLL binding motifs of E6AP and MAML1 [34].

Relatively few E6 proteins have been characterized for their associations with E6AP compared to MAML1. In this study, we have expressed 45 different E6 proteins from 21 animal and human genera. We have determined the preferential association of each E6 protein with either E6AP or MAML1, and performed functional assays for either transcriptional repression of MAML1 or the stimulation of E6AP degradation by E6 in vivo. The results clearly divide genera of papillomaviruses that express E6 proteins into those that physically and functionally target MAML1 and not E6AP, and those that physically and functionally target E6AP and not MAML1. We further make mutations in E6AP and thereby demonstrate that Alpha genus E6 proteins have a second function separate from binding to the LXXLL motif of E6AP that is required to efficiently stimulate E6AP degradation, demonstrating that the ability of Alpha genus E6 proteins to confer E6AP dependent degradation of cellular proteins is not triggered solely by association with an LXXLL motif on E6AP. This analysis delineates a profound functional split among papillomavirus genera and suggests that a functional grouping of papillomaviruses into those that target Notch signaling through interaction with MAML proteins, and those that target cellular protein degradation through association with E6AP provides insight into papillomavirus taxonomy.

## Results

**Fig 1:**
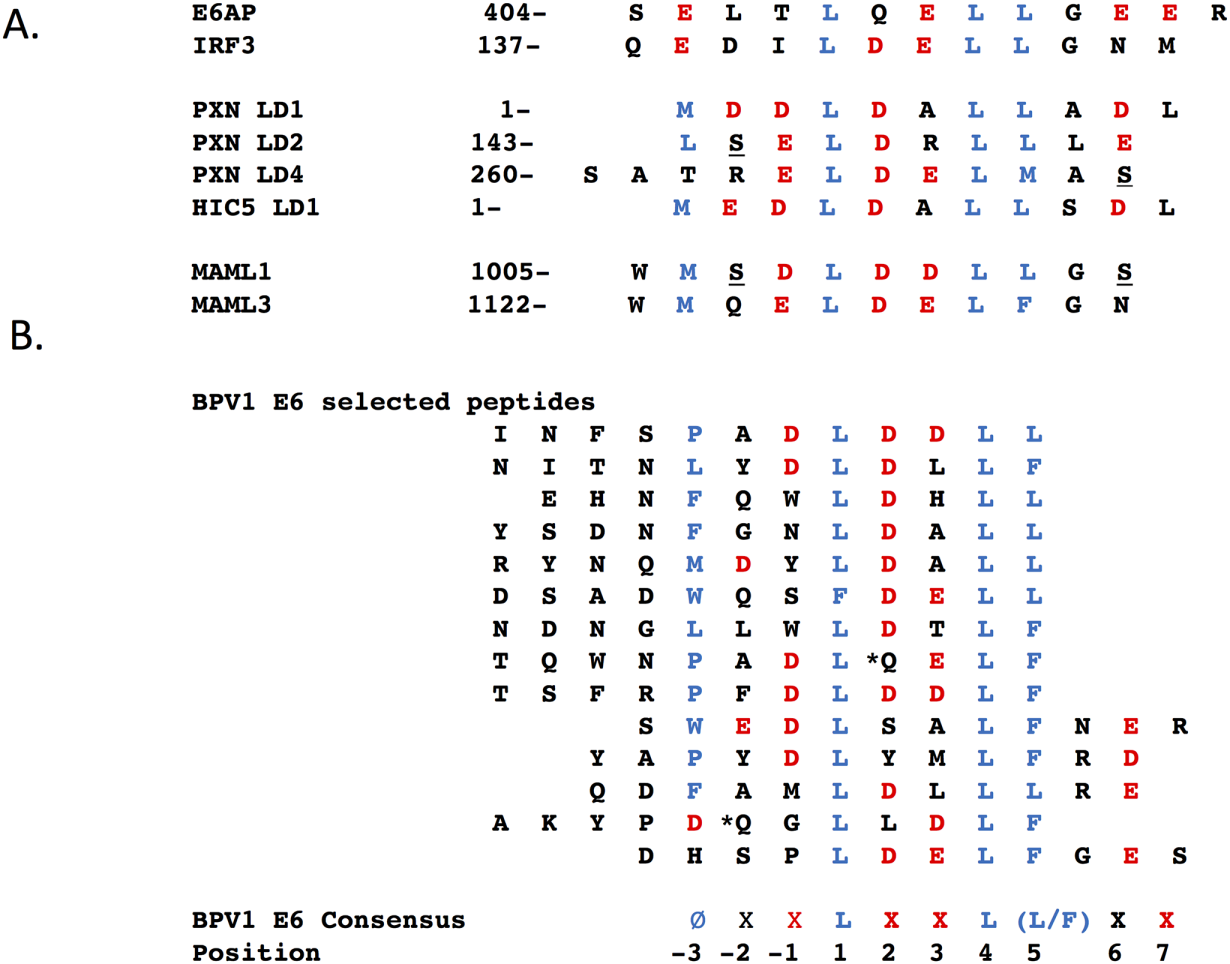
LXXLL peptide sequences that interact with BPV1 E6 and HPV16 E6. A. LXXLL peptide sequences within the indicated cellular proteins that serve as docking sites for Alpha genus HPV16 E6 (top two peptides, E6AP (NP_000453.2), and IRF3, (NP_001184051.1); and Delta genus BPV1 E6, PXN (XP_010812366.2), HIC5 (NP_001035919), MAML1 (NP_055572.1) and MAML3 (NP_061187.2). Potentially phosphorylated sites (underlined) were identified at PhosphositePlus.org [107], but only PXN pS-273 is confirmed in a publication [108]. Hydrophobic residues are colored blue, and acidic residues are in red. B. The bottom grouping of 14 peptide sequences were selected for BPV1 E6 association from a phage display expression library as detailed in the methods. Amino acids with an associated asterix-encoded a stop codon at that position are suppressed by supE44 suppressor tRNA from amber to glutamine in the E.coli strain used to propagate the M13 phage. At the bottom, a consensus sequence for BPV1 E6 associated LXXLL peptides and the numbering scheme used in this manuscript are shown.

Fig 1 shows LXXLL motifs from cellular proteins that associate with E6 proteins. The LXXLL motifs from E6AP and IRF3 are bound by some Alpha genus E6 proteins [27, 38, 39]. PXN and HIC5 motifs are bound by Delta genus BPV1 E6 [31], and MAML1 by Delta, Mu, Pi and Beta genus HPV E6 proteins [34–37, 40]. Although there are clear similarities among these few known LXXLL motifs, the range of possible binding sites from a very large set of random possible ligands has not been explored.

In order to explore LXXLL binding motifs in an unbiased way, BPV1 E6 was expressed and used to select peptide interactors from a very large (> 10^12^ independent clones) phage display library where random sequence 12-mer peptides were displayed as fusions to M13 gene-III. After five rounds of selection, 25 plaques were sequenced revealing 14 unique sequences; all the unique sequences are shown in Fig 1 and aligned where a ØXXØØ-like sequence (Ø representing a hydrophobic side chain) could be discerned; a consensus sequence and position numbering are at the bottom of Fig 1. The core ØXXØØ was observed in all 14 peptides, as LXXLL six times and as LXXLF eight times. Notably, a clear selection for a hydrophobic residue at position -3 upstream of the LXXL/F was observed in 12 of 14 selected peptides, making this the second most prevalent selected feature, followed by the presence of acidic side chains at positions two (10 of 14 peptides), positions -1 and 3 (found in six of 14 peptides) and position 7 (four of 14 peptides). A consensus for preferred BPV1 E6-bound peptides from this experiment is thus ØX(D)L(D/E)(D/E)L(L/F)X(D/E). This consensus is quite similar to the LXXLL motifs in cellular proteins that bind BPV1 E6, shown in Fig 1A.

**Fig 2:**
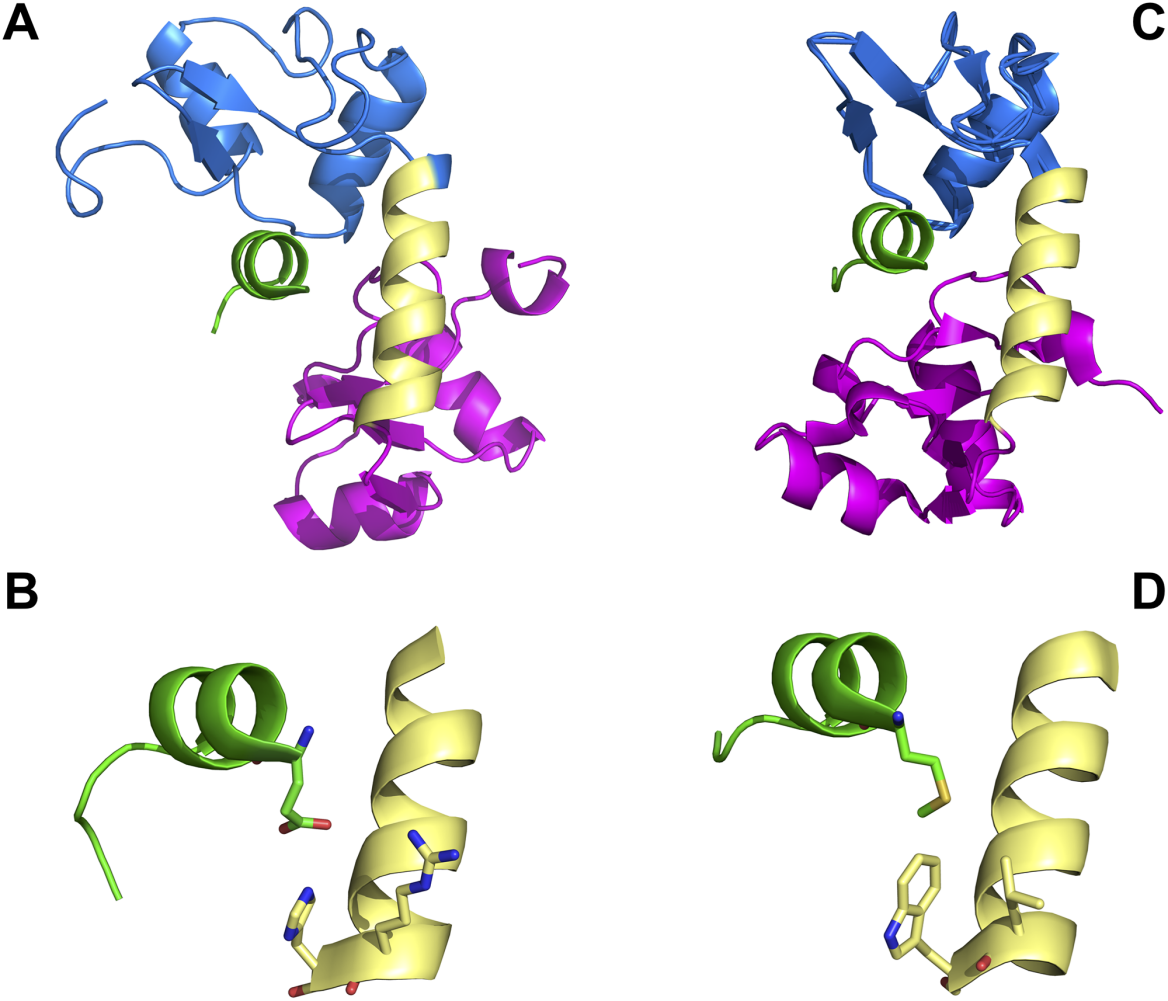
Contact residues in HPV16 E6 and BPV1 E6 that distinguish between E6AP interaction and MAML1 interaction. A. HPV16 E6 structure (PDB file 4GIZ [41]) showing the amino-terminal zinc structured domain in blue, the connecting helix in yellow and the carboxy-terminal zinc-structured domain in pink) in association with the LXXLL peptide of E6AP (green) where the amino-terminal position -3 is facing the viewer. B. A close-up detail from part A of the HPV16 E6 –E6AP structure showing the interaction between position -3 of the E6AP LXXLL peptide (glutamic acid) with 16E6 amino acids R77 and H78. C. The structure of BPV1 E6 in association with the LXXLL peptide of paxillin (PDB file 3PY7 [41]) in similar orientation to the structure in part A. D. A close-up detail from part C of the BPV1 E6 structure showing the interaction between position -3 of the PXN LXXLL peptide (methionine) with BPV1 E6 amino acids L64 and W65.

The strong prevalence of a hydrophobic side chain at position -3 in BPV1-bound phage-selected peptides contrasts with the LXXLL peptides found in E6AP and IRF3 that interact with high-risk Alpha genus HPV16 E6, where position -3 in both LXXLL peptides is a glutamic acid. The crystal structures of BPV1 E6 and HPV16 E6 bound to LXXLL motifs of PXN and E6AP respectively have been solved, and show a conserved overall fold and mode of interaction between the two E6 proteins and their respective LXXLL peptides [41] [42]. In the BPV1 E6 structure, the amino acid side chain M1 of the bound PXN-derived LXXLL peptide resides within a hydrophobic pocket comprised of E61, L64 and W65 and the side chain of R116 (that interacts with E61 of BPV1 E6 and D5 of the PXN LXXLL peptide); in the HPV16 E6–E6AP structure, the analogous E1 residue of the E6AP LXXLL peptide interacts with the analogous E6 residues of HPV16 E6 (S74, H78, R77 and R129) [41]. The overall structure and detail illustrating this is shown in Fig 2. The strong selection for a hydrophobic amino acid side chain at position -3 among the BPV1 E6 phage-selected peptides illuminates the important role of this position when E6 proteins discriminate between interactions with the LXXLL peptide derived from E6AP, compared to peptides from PXN, HIC5, MAML1 or MAML3. Interestingly, BPV1 E6 selected peptides with an LXXLF sequence as well as LXXLL (Fig 1B). LXXLF is found in MAML3, and MAML3 as well as MAML1 were found in association with BPV1 E6 [37].

Given that analogous amino acids in HPV16 E6 and BPV1 E6 served similar structural functions in binding to position -3 of their respective LXXLL peptides, we wondered if examination of these contact amino acids in diverse E6 proteins might predict the preference of a given E6 protein for either E6AP or MAML1. Within the Alpha genus, precise conservation of these contact residues was overall modest. For the four amino acids with closest contacts with position -3 of E6AP, S74 was poorly conserved, R77 was highly conserved and H78 was moderately conserved (present in 52/78 Alpha genus E6 proteins); interestingly, H78 was replaced by a hydrophobic residue (similar to that seen in BPV1 E6) in 26/78 Alpha E6 sequences, and the remaining contact residue, R129, was poorly conserved (a multiple sequence alignment of the Alpha genus E6 proteins is shown in S1 Fig). Thus, examination of the contact residues of Alpha genus E6 proteins found they were not sufficiently conserved and could not indicate that E6 proteins would interact with E6AP and/or possibly an additional LXXLL binding site such as on MAML1.

As noted above, the Beta genus HPV E6 proteins interact with the LXXLL motif of MAML1. A similar examination of the predicted contact residues between E6 and position M1 of the MAML1 LXXLL was more consistently predictive, but not completely. The position analogous to HPV16 E6 S74 was highly conserved as a glutamic or aspartic acid, the position of HPV16 E6 R77 was not conserved, but the position analogous to HPV16 H78 was hydrophobic as expected (being either phenylalanine or tyrosine in 48/50 examined Beta genus sequences), and the R129 position of HPV16 E6 (analogous to R116 of BPV1 E6) was completely conserved in the Beta and Gamma genus E6 proteins (S2 and S3 Figs).

**Fig 3:**
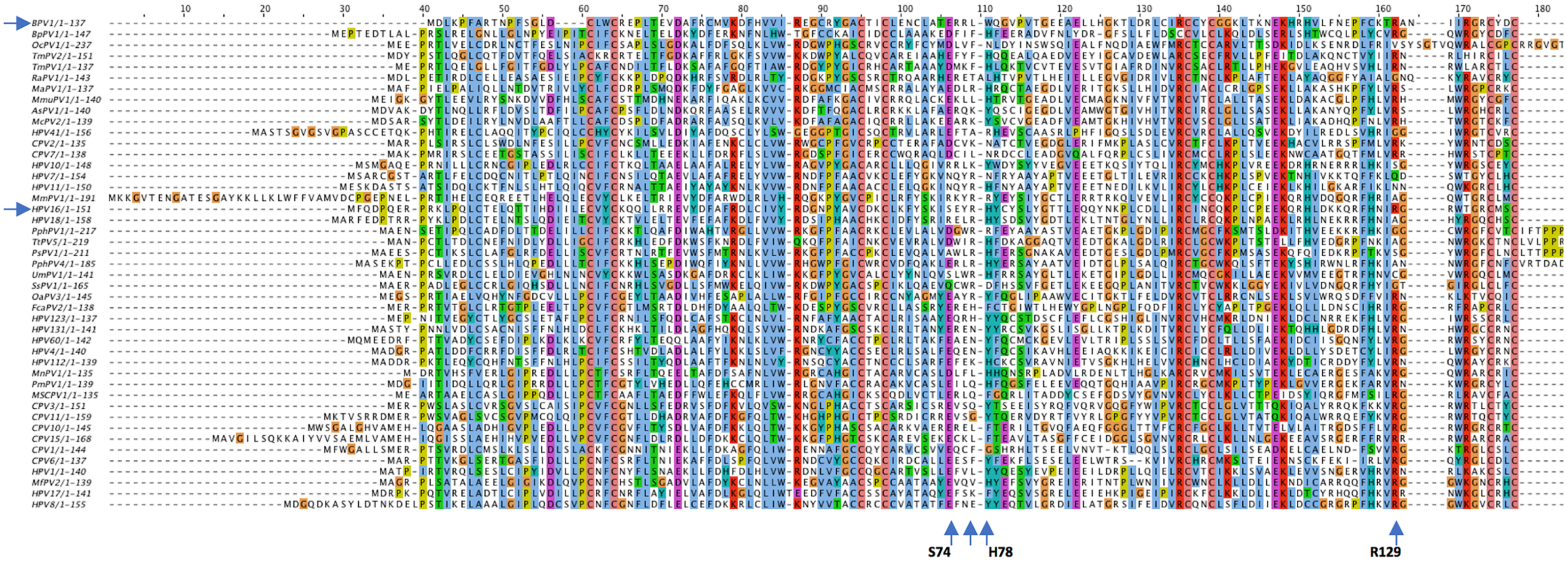
Multiple sequence alignment of the studied E6 protein set. A protein multiple sequence alignment was performed using the MUSCLE program [103], and the output then entered into Jalview2 [109] to generate the ClustalX colorized image. The four upward-pointing arrows at the bottom of the alignment indicate the positions of HPV16 E6 amino acids that interact with position -3 of the E6AP LXXLL motif and BPV1 E6 interaction with PXN as shown in Fig 1. The two horizontal arrows on the left denote the positions of BPV1 E6 and HPV16 E6 in the alignment.

The heterogeneity in the E6 contact residues for interaction with position -3 of the LXXLL binding sites suggested the possibility that some E6 proteins within a genus might have multiple different LXXLL interaction targets, perhaps interacting with both E6AP and MAML proteins as has been recently suggested for Gamma genus HPV197 E6 [43]. We wished to determine if within or between papillomavirus genera, E6 proteins had distinct binding and functional preferences for either E6AP or MAML family proteins. We synthesized 45 different E6 genes where the E6 protein shared the canonical E6 structure of two zinc structured domains connected by an alpha helix, and one E6 protein (OcPV1) where an additional zinc structured domain is found at the carboxy-terminus (as is present in Shope Papillomavirus E6). Table 1 shows the tested E6 proteins and their host species. A multiple sequence analysis of the 45 tested E6 proteins is shown in Fig 3, and shows the amino acid positions in HPV16 and BPV1 E6 amino acids that interact with position -3 of the E6AP or MAML1 LXXLL peptides. The position of the tested E6 proteins within a phylogeny map of animal E6 proteins and a subset of HPV E6 proteins is shown in S4 Fig.

**Table 1:** 

Host Common name	Full name	Symbol	genera	Accession no.	Associated Lesion(s)	Reference
Domestic cow	Bos taurus papillomavirus 1	BPV1	Deltapapillomavirus	X02346	Cutaneous fibropapilloma, fibrosarcoma	[44]
Dmestic sheep	Ovies aries papillomavirus 3	OaPV3	Dyolambdapapillomavirus	FJ796965.1	Squamous carcinoma, nl skin	[17]
Domestic pig	Sus scrofa domesticus papillomavirus 1	SsPV1	Dyodeltapapillomavirus	EF395818	Healthy skin	[45]
Domestic dog	Canis familiaris papillomavirus 2	CPV2	Taupapillomavirus 1	AY722648	foot papillomma, squamous cell carcinoma	[46]
Domestic and	Canis familiaris oral papillomavirus	CPV1	Lambdapapillomavirus	D55633.1	Oropharyngeal papilloma	[47]
Domestic dog	Canis familiaris papillomavirus 3	CPV3	Chipapillomavirus	DQ295066.1	Squamous carcinoma, EV -like disease	[48]
Domestic dog	Canis familiaris papillomavirus 6	CPV6	Lambdapapillomavirus	FJ492744.1	Mucosal throat papilloma	[49]
Domestic dog	Canis familiaris papillomavirus 7	CPV7	Taupapillomavirus	FJ492742.1	In situ squamous cell carcinoma	[49]
Domestic dog	Canis familiaris papillomavirus 10	CPV10	Chipapillomavirus	JF800657.1	Pigmented cutaneous plaque	[50]
Domestic dog	Canis familiaris papillomavirus 11	CPV11	Chipapillomavirus	JF800658.1	Pigmented cutaneous plaque	[51]
Domestic dog	Canis familiaris papillomavirus 15	CPV15	Chipapillomavirus	JX899359.1	N.R.^2^	N.A.^1^
Domestic cat	Felis domesticus papillomavirus 2	FcaPV2	Dyothetapapillomavirus	EU796884.1	Cutaneous carcinoma in situ	[52]
Human	Human Papillomaviirus type 1	HPV1	Mupapillomavirus	NC_001356.1	Plantar papilloma	[53]
Human	Human Papillomaviirus type 4	HPV4	Gammapapillomavirus	X70827.1	Plantar papilloma	[54]
Human	Human Papillomaviirus type 7	HPV7	Alphapapillomavirus	X74463.1	Butcher's wart	[55]
Human	Human Papillomaviirus type 8	HPV8	Betapapillomavirus	NP_068490	Epidermodysplasia Verruciformis	[56]
Human	Human Papillomaviirus type 10	HPV10	Alphapapillomavirus	NC_001576	flat warts,	[57]
Human	Human Papillomaviirus type 11	HPV11	Alphapapillomavirus	M14119	Laryngeal papilloma	[58]
Human	Human Papillomaviirus type 16	HPV16	Alphapapillomavirus	NC_001526	Cervical carcinoma	[59]
Human	Human Papillomaviirus type 17	HPV17	Betapapillomavirus	JN211195	Epidermodysplasia Verruciformis	[60]
Human	Human Papillomaviirus type 18	HPV18	Alphapapillomavirus	NC_001357	cervical cancer	[61]
Human	Human Papillomaviirus type 41	HPV41	Nupapillomavirus	NC_001354	fascial papilloma	[62]
Human	Human Papillomaviirus type 60	HPV60	Gammapapillomavirus	U31792	plantar cyst	[63]
Human	Human Papillomaviirus type 112	HPV112	Gammapapillomavirus	NC_012486.1	condyloma	[64]
Human	Human Papillomaviirus type 123	HPV123	Gammapapillomavirus	GQ845445.1	undescribed	[65]
Human	Human Papillomaviirus type 131	HPV131	Gammapapillomavirus	NC_014954.1	cutaneous	N.A.
Polar bear	Ursus maritimus papillomavirus 1	UmPV1	Omegapapillomavirus	EF536349.1	oral tongue papilloma	[66]
Harbor porpoise	Phocoena phocoena papillomavirus 1	PphPV1	Omikronpapillomavirus	GU117621.1	Penile papilloma	[67]
Harbor Porpoise	Phocoena phocoena papillomavirus 4	PphPV4	Dyopipapillomavirus	GU117623.1	Penile papilloma	[67]
Burmeister's porpoise	Phocoena spinipinnis papillomavirus 1	PsPV1	Omikronpapillomavirus	AJ238373.1	Genital papilloma	[68]
Bottle-nose dolphin	Tursiops truncatus papillomavirus 5	TtPV5	Omikronpapillomavirus	JN709470.1	Penile papilloma	[69]
Florida Manatee	Trichechus manatus latirostris papillomavirus	TmPV2	Rhopapillomavirus	JN709473	N.R.	N.A.
Florida Manatee	Trichechus manatus latirostris	TmPV1	Rhopapillomavirus	AY609301	Cutaneous papilloma	[70]
Bent-wing bat	Miniopterus schreibersii papillomavirus 1	MscPV1	unclassified	JQ814848	Pharyngeal swab or anal swab	[71]
Egyptian fruit bat	Rousettus aegyptiacus papillomavirus 1	RaPV1	Psipapillomavirus	DQ366842.1	Cutaneous squamous carcinoma	[72]
Brush-tailed bettong	Bettongia penicillata papillomavirus 1	BpPV1	Dyokappapapillomavirus	GU220391.1	Cutaneous papilloma	[73]
New Zealand rabbit	Oryctolagus cuniculus papillomavirus 1	OcPV1	Kappapapillomavirus	AF227240.1	Oral and genital papillomas	[74]
Crab-eating macaque	Macaca fascicularis papillomavirus 2	MfPV2	Betapapillomavirus	GU014531.1	Cutaneous papilloma	[75]
Rhesus macaque	Macaca mulatta papillomavirus 1	MmPV1	Alphapapillomavirus	M60184.1	Mucosal genital carcinoma	[76]
Wood mouse	Apodemus sylvaticus papillomavirus 1	AsPV1	Pipapillomavirus	HQ625440	Normal skin (ear)	[77]
Natal multi-mammate mouse	Mastomys natalensis papillomavirus 1	MnPV1	Iotapapillomavirus	U01834	Cutaneous papilloma	[78]
Southern multimammate mouse	Mastomys coucha papillomavirus 2	McPV2	Pipapillomavirus	DQ664501	Skin carcinoma	N.A.
Syrian golden hamster	Mesocricetus auratus papillomavirus 1	MaPV1	Pipapillomavirus	E15111	Oral papilloma	N.A.
Laboratory mouse	Mus musculus papillomavirus 1	MmuPV1	Pipapillomavirus	GU808564	Cutaneous papilloma	[79]
Deer mouse	Peromyscus maniculatus papillomavirus 1	PmPV1	unclassified	JF755418	Feces	[80]

N.R., not reported

**Fig 4:**
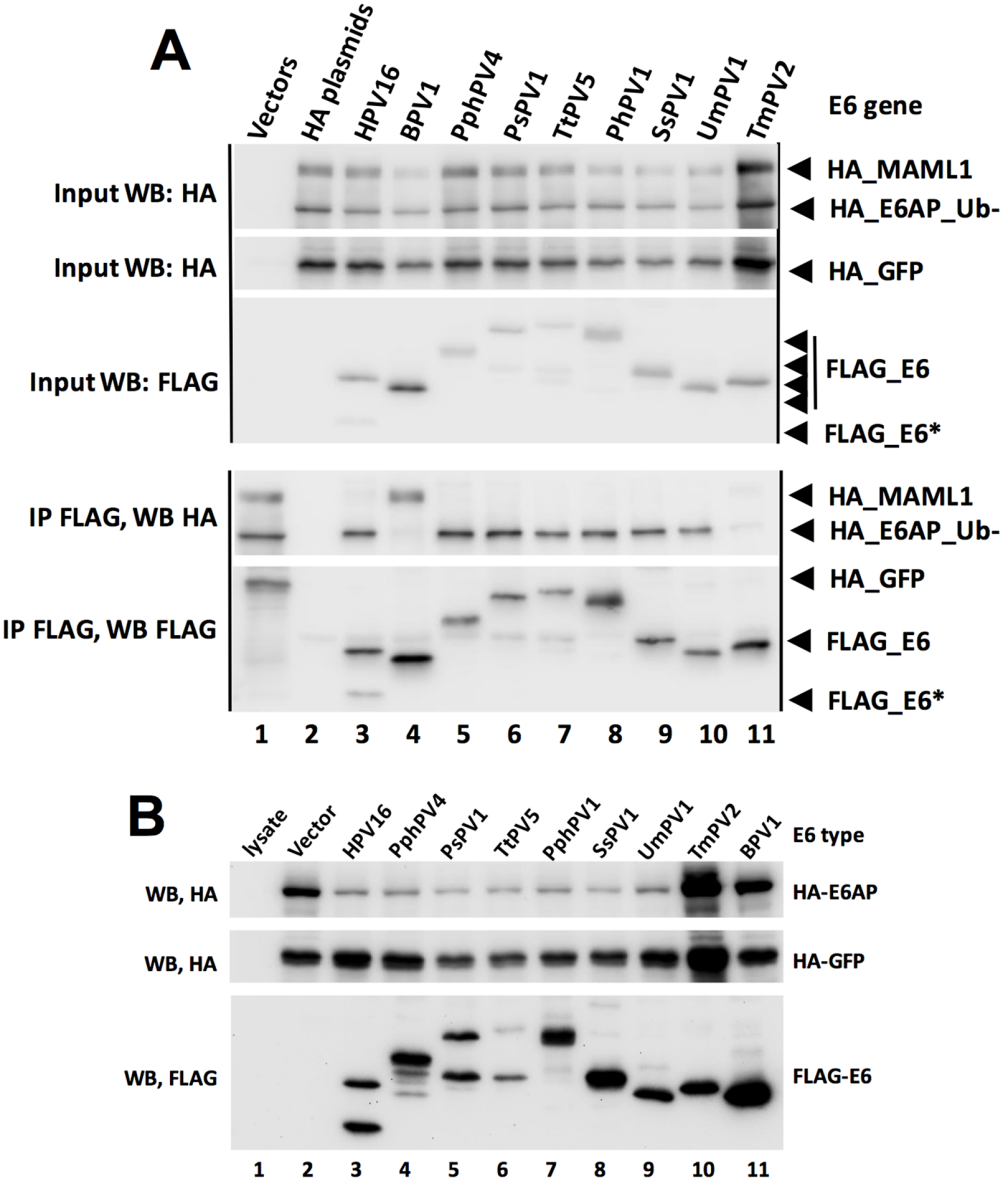
Preferential physical and functional discrimination between E6AP and MAML1 by E6 proteins. A. Preferential association of E6 proteins for either E6AP or MAML1. HA-tagged E6AP_Ub-, MAML1 and GFP expression plasmids were co-transfected with the indicated FLAG-tagged E6 expression plasmids into 293T cells and harvested 18 hrs. later in 0.5X IPEGAL lysis buffer as described in the methods. Western blots of input samples are clustered at the top and FLAG immune precipitated samples at the bottom, except that lane 1 at the bottom cluster of blots is an input sample, same as lane 2 in the top cluster of blots. Input was 4% of the immune precipitated sample size. E6* is a spliced and truncated E6 variant. B. Alpha genera and selected genus E6 proteins reduce the expression of E6AP upon co-expression. HA-tagged E6AP, HA-GFP and the indicated FLAG-tagged E6 proteins were co-transfected into 293T cells and cells were lysed in SDS sample buffer 18 hrs. later.

**Fig 5:**
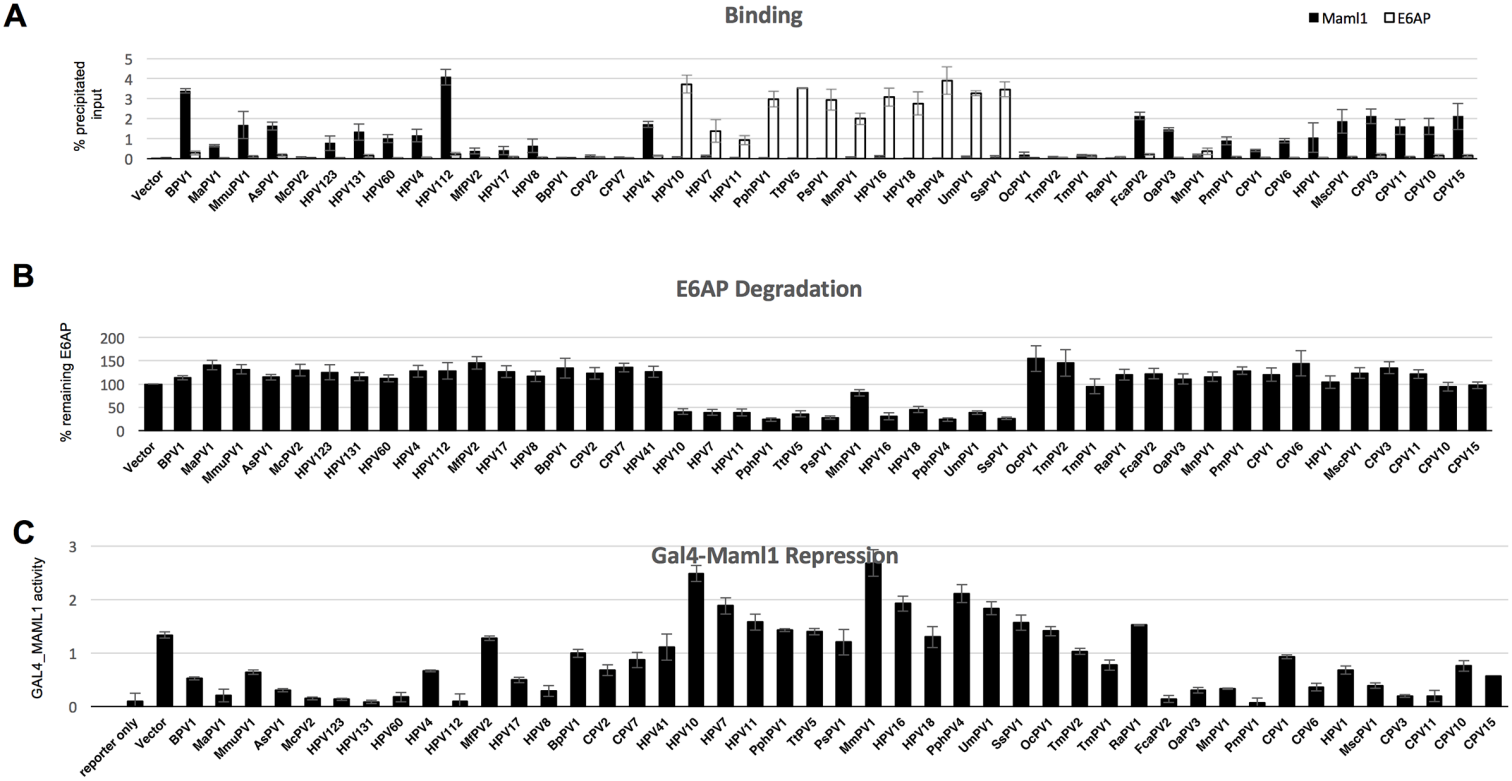
E6 proteins discriminate in functional and physical interaction between E6AP_Ub- and MAML1. **A**. Comparative binding of E6 proteins to MAML1 or E6AP. Quantitation of input HA-tagged E6AP_UB- and HA-tagged MAML1 were immune precipitated by anti-FLAG-E6 proteins as shown in Fig 4 and S5 Fig, and triplicate experiments quantified. Black bars represent MAML1 precipitation and white bars E6AP precipitation. The vertical axis identifies the percentage of input protein precipitated. The averages of three separate experimental replicates with standard deviations is shown. B. HA-E6AP was co-transfected together with the indicated FLAG-tagged E6 proteins and degradation of E6AP quantified relative to vector transfected HA-E6AP, and normalized to a co-transfected HA-GFP to adjust for differences in transfection efficiency. Results are averages with standard deviations from three independent experimental replicates. C. E6 proteins that associate with MAML1 repress GAL4-MAML1 transcriptional activation. GAL4-MAML1, GAL4-luciferase reporter, pCMV-renilla internal transfection control and empty vector or the indicated E6 proteins were co-transfected into 293T cells and harvested for luciferase and renilla assay 18 hrs. later. Results are the average and standard deviation of four independent experiments with duplicate samples in each experiment, and normalized to co-transfected renilla activity.

To test the preference of each E6 protein for either MAML1 or E6AP association, we co-expressed FLAG-E6 together with an excess of both HA-E6AP_Ub- (Ub- means mutated in ubiquitin ligase activity) and HA-MAML1, such that HA-E6AP_Ub- and HA-MAML1 were similar in abundance, and less than 10% of the input HA-tagged proteins were immunoprecipitated by FLAG-E6. We reasoned that overexpression of the HA-tagged E6-binding targets compared to E6 would promote competition between HA-E6AP and HA-MAML1 for the available E6, and thereby serve as an indication of the preference of each E6 protein for either MAML1 or E6AP_Ub-. Fig 4A shows a typical experimental result with nine different E6 proteins, some of which the preference for E6AP or MAML1 association was previously described [34, 81]. Representative western blots for the remaining E6 proteins are shown in S5 Fig. These experiments were repeated to obtain quantitative results with error which is shown in Fig 5A. Typically, E6 proteins showed a clear preference for either E6AP or MAML1 association. In some cases, an E6 protein clearly discriminates in binding between E6AP compared to MAML1, but that discrimination did not reach statistical significance in the three experimental replicates (such as HPV11, 17, and 8). Typically, those E6 types had low levels of association to both E6AP and MAML1; low expression levels reduce net levels over background and therefore increases the associated error. Even in those cases, a preference of binding to either E6AP or MAML1 is clear on the multiple western blots, allowing a qualitative assessment of binding preference to be made.

We desired to correlate the physical association of E6 with E6AP to an in vivo biological function. We and others had previously observed that overexpression of E6 together with E6AP WT results in reduced expression of E6AP because of E6AP ubiquitin ligase activity and proteasome mediated degradation; in contrast, BPV1 E6 does not stimulate E6AP degradation [27, 82]. Each E6 protein was co-transfected with E6AP WT, and the reduction in E6AP expression upon co-expression with E6 was ascertained by western blot (Fig 4B and S6 Fig) with quantitation after three experimental replicates shown in Fig 5B. As expected, E6 proteins from the Alpha genus (HPV types 7, 10, 11, 16, 18 and MmPV1) that associated preferentially with E6AP also stimulated E6AP degradation. While MmPV1 (also known as RhPV1 for Rhesus Papillomavirus Type 1) was less active than other Alpha proteins in stimulating E6AP degradation, it did so significantly and also promoted degradation of p53 in an E6AP-dependent manner (S7 Fig). Other genera whose E6 proteins preferentially associated with E6AP also stimulated E6AP degradation, including Omega (UmPV1), DyoDelta (SsPV1), Omikron (PhPV1, TtPV5, PsPV1), and Dyopipa (PhPV4).

To correlate the association of E6 proteins with MAML1 to an in vivo biological activity, we co-transfected the test set of E6 proteins together with a GAL4_MAML1 fusion, and measured the repression of a GAL4 responsive luciferase reporter as previously described [34]. Fig 5C shows that E6 proteins that preferentially associate with MAML1 also repress GAL4_MAML1 transcriptional activation. Some E6 proteins that weakly associated with MAML1 by IP/WB still significantly repressed GAL4-MAML1 activity (HPV8, HPV17, HPV1, HPV123, MnPV1, MfPV2, and McPV2), indicating that some of the weak interactions with MAML1 in our binding assay were biologically significant. Only HPV41 E6 strongly associated with MAML1 but failed to significantly repress GAL4-MAML1 transcriptional activation (Fig 5A and 5C).

**Fig 6:**
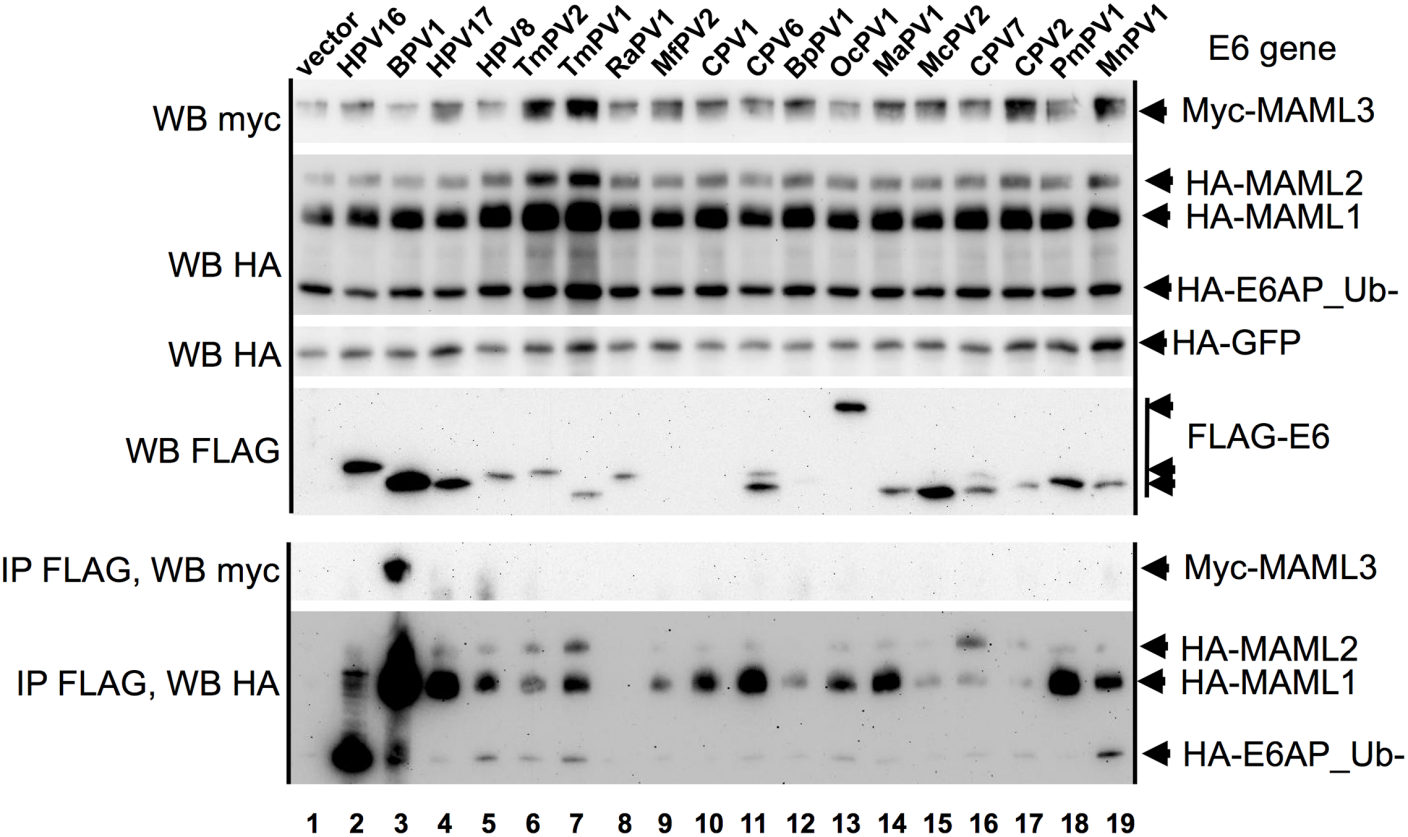
Preferential association of MAML1 compared to MAML2 or MAML3 with E6 proteins. 293T cells were co-transfected with myc-MAML3, HA-MAML1, HA-MAML2, HA-E6AP_Ub-, HA-GFP, and the indicated FLAG-E6 proteins. 18 hrs. later cells were lysed, an input aliquot of lysate removed, and the remainder of the clarified lysate immune precipitated with FLAG antibody beads as described in the methods. One of two replicated experiments are shown.

Some E6 proteins that did not clearly associate with either E6AP or MAML1 also failed to repress either GAL4_MAML1 activity or reduce E6AP expression levels (TmPV1, TmPV2, RaPV1, BpPV1, and OcPV1). Examination of the LXXLL motif at the carboxyl-terminus of MAML1, 2, or 3 reveals significant differences between the three LXXLL motifs (S1 Table). We therefore tested the E6 proteins that interacted poorly with E6AP and MAML1 for their possible interaction with either MAML2 or MAML3 by co-expression and co-immunoprecipitation. Only BPV1 E6 co-immunoprecipitated MAML3, which is consistent with the prior finding of BPV1 E6 in association with MAML3 from the Howley lab [37]. Only CPV7 E6 showed preferential interaction with MAML2 compared to MAML1 (Fig 6). Thus overall, there was a strong preference for MAML1 association with E6 from many divergent host species.

The LXXLL motifs of E6AP, MAML2, and MAML3 were quite conserved among the host species in our test set of E6 proteins, however several host species had one or more amino acid changes in the LXXLL motif of MAML1 (S1 Table). To test the relevance of host-specific changes in the MAML1 LXXLL motifs, we mutated the LXXLL motifs in human MAML1 into the LXXLL motifs of rodent or canine species. These chimeric MAML1 molecules were co-transfected in comparison with human MAML1 to determine if species preference for cognate LXXLL motifs accounted for the limited associations of these E6 proteins with human MAML1 observed in Fig 5. Surprisingly, all tested rodent and canine E6 proteins showed similar preference for human or their cognate species MAML1 chimeric protein except MmaPV1, which surprisingly, showed a modest preference for human MAML1 compared to the human-rodent MAML1 chimera (S8 Fig).

**Fig 7:**
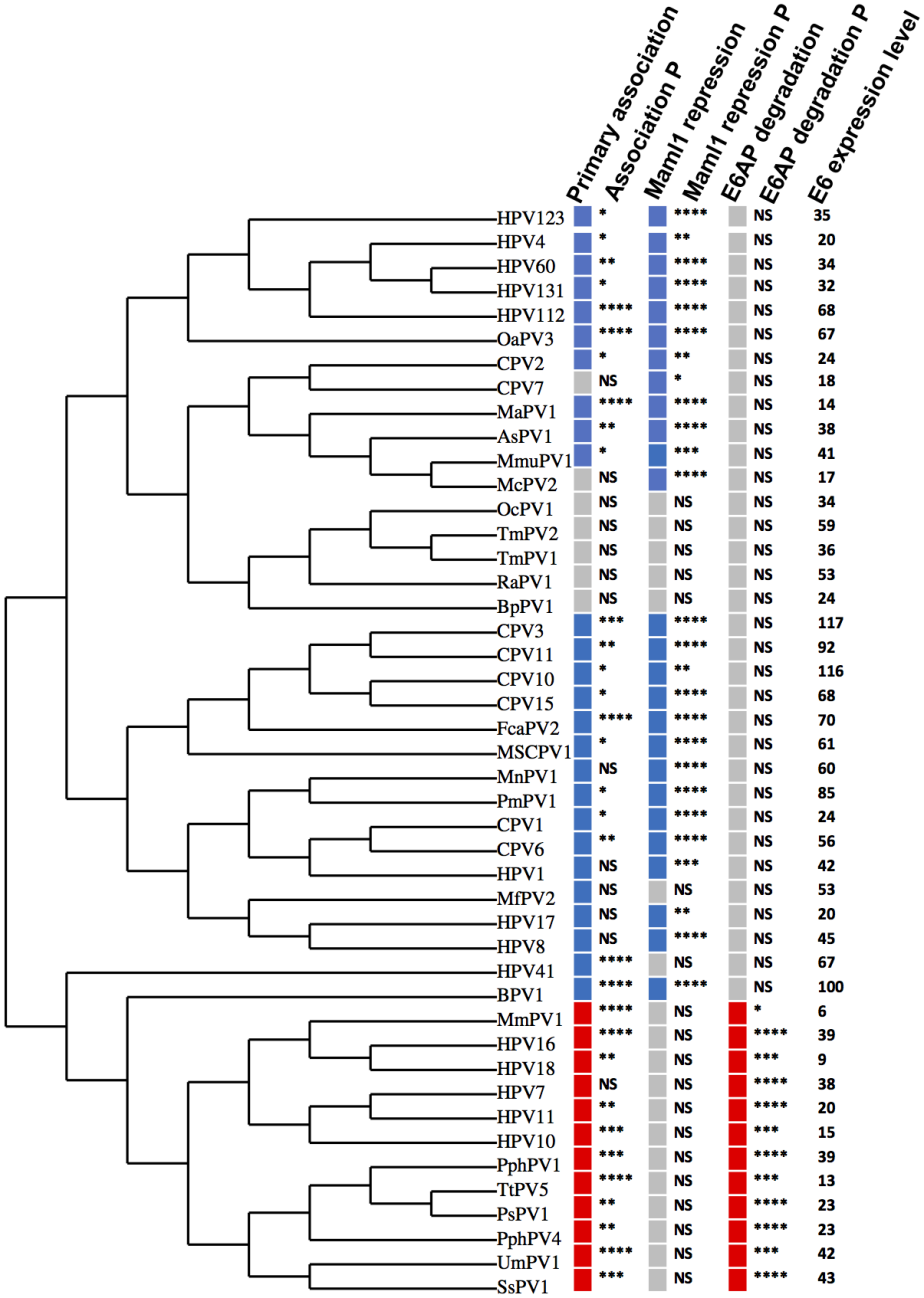
E6 proteins that physically and functionally interact with either E6AP or MAML1 cluster phylogenetically. The cladogram is derived from a multiple sequence alignment using the program “MUSCLE” [103], clustering phylogenetically using PhyML [105], and tree rendering with TreeDyn [106]. For physical association by immune precipitation, blue denotes MAML1, red E6AP, and grey denotes neither MAML1 nor E6AP. P denotes the statistical significance of the association as determined from the quantitation of at least three independent experiments of quantified western blot data or four independent Luciferase assays analyzed by Student’s t test (NS, not significant; * <0.05; ** < 0.01; *** < 0.001; **** < 0.0001). For some samples, low-level MAML1 was consistently observed upon co-immune precipitation and so colored, even though statistical significance was not reached. For MAML1 repression, blue denotes repression of GAL4-MAML1, and grey no repression. E6-induced E6AP degradation is shown in red color, and lack of degradation in grey. E6 expression level is from a representative experiment and is normalized to the expression of BPV1 E6, set at 100.

Using the multiple sequence alignment (Fig 3), a cladogram was generated to illustrate the clustering of the tested E6 proteins by sequence relatedness. Qualitative binding data and statistical significance for the physical association of each tested E6 protein with MAML1 and E6AP is shown, compared together with E6AP degradation and GAL4-MAML1 repression to illustrate clustering of preferential interactions with biological functions (Fig 7). Several observations were apparent.

First, E6 proteins from most tested genera preferentially associated with MAML1 compared to E6AP. Where the physical association with MAML1 was statistically significant, in all but one case (HPV41) GAL4_MAML1 activity was significantly repressed. All the tested Gamma and Nu (HPV41) genera E6 proteins preferentially associated with MAML1 and all except HPV41 E6 significantly repressed GAL4_MAML1.

Second, E6 proteins that bound neither E6AP nor MAML1 did not cluster with E6 proteins that did associate with MAML1 or E6AP: OcPV1, TmPV1, TmPV2, RaPV1, and BpPV1. RaPV1 bound both MAML1 and E6AP at low levels, but neither decreased E6AP expression nor repressed GAL4_MAML1 transcriptional activation. The characterization of our test set of E6 proteins within the larger set of all animal E6 proteins and a subset of HPV E6 proteins is shown in S9 Fig.

Third, E6 proteins that preferentially associated with E6AP compared to MAML1 both reduced E6AP expression levels and failed to repress GAL4_MAML1 transcriptional activation. Phylogenetically clustered together with the Alpha genera E6 proteins are E6 proteins from Omega, Omikron, DyoDelta, and Dyopipa genera. Since only E6 proteins that preferentially associated with E6AP were able to reduce its expression level, it is unclear from these experiments if it was the docking of an E6 protein to the LXXLL motif of E6AP that triggers degradation, or if a second property of E6, subsequent to the initial and required binding to the LXXLL motif, was also required to initiate degradation.

**Fig 8:**
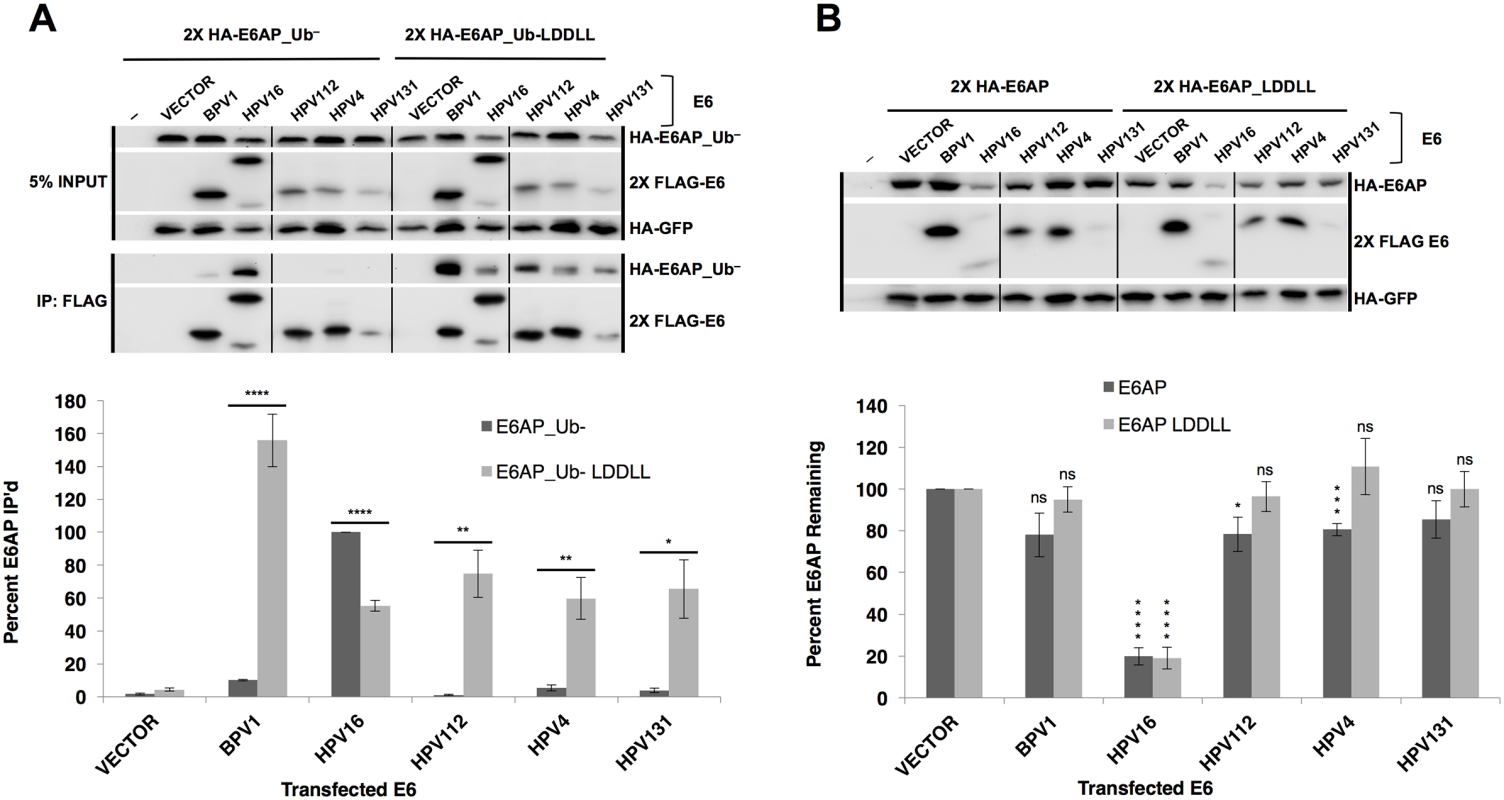
Binding of Delta or Gamma genera E6 proteins to E6AP is not sufficient to initiate E6AP degradation. (A) HA-E6AP_Ub- or HA-E6AP_Ub- LDDLL (E6AP where the LXXLL motif was mutated to the MAML1 LXXLL amino acid sequence) were co-transfected with the indicated E6 protein and HA-GFP in 293T cells. Cells were lysed 18 hours post-transfection in 0.5X IPEGAL lysis buffer. E6AP_Ub- does not immunoprecipitate with Delta or Gamma E6 proteins (BPV1, HPV112, HPV4, and HPV131) but E6AP_Ub- LDDLL immunoprecipitates with both Delta and Gamma genera E6 proteins. Alpha genera HPV16 E6 immunoprecipitates both E6AP_Ub- and E6AP_Ub- LDDLL, but significantly less of the MAML-like E6AP. Vertical black line indicates removal of an irrelevant sample. Quantification normalized to HPV16 E6 sample (= 100%). n = 4, * < 0.05; ** < 0.01; **** < 0.0001 by Student’s t-test. (**B**) Binding of E6 to LXXLL of E6AP is insufficient to stimulate E6AP degradation. Although Alpha, Gamma, and Delta E6 proteins bind to E6AP LDDLL (panel A), only Alpha E6 stimulates E6AP degradation. 293T cells were transfected with indicated E6 protein, HA-GFP, and either HA-E6AP or HA-E6AP LDDLL. 18 hours post-transfection, cells were lysed in 1X SDS sample buffer and E6AP expression determined by western blot. Vertical black line indicates removal of an irrelevant sample. Quantified samples normalized to HA-GFP and vector transfected cells. n = 4, * < 0.05; *** < 0.001; **** < 0.0001 by Student’s t-test.

To address this question, an E6AP molecule was constructed where the LXXLL binding motif of E6AP (ELTLQELLGEE) was mutated into the LXXLL motif of MAML1 (MSDLDDLLGS, the new E6AP mutant is termed E6AP_LDDLL). While BPV1 E6, and E6 proteins from HPV types 112, 4, and 131 (Gamma genus) preferentially associate with MAML1 and not E6AP (Fig 5), all interacted robustly with E6AP_Ub-_LDDLL (Fig 8A). Interestingly, HPV16 E6 bound both E6AP_Ub- and to a lesser extent, E6AP_Ub-_LDDLL, despite poor interaction with MAML1 (Figs 5 and 8A). HPV16 E6 still initiated degradation of both E6AP WT and E6AP_LDDLL. In contrast, BPV1 and HPV types 4, 112, and 131 E6 proteins did not interact with E6AP_Ub-, but interacted robustly with E6AP_LDDLL, even more than did HPV16 E6. However, despite robust interaction with E6AP_LDDLL, BPV1, and HPV types 4, 112, and 131 E6 did not reduce the expression of E6AP_LDDLL (Fig 8B). This demonstrates that while association of E6 with E6AP is required to initiate E6AP degradation, some difference in the binding or an additional property of Alpha genera E6 beyond simply associating with the LXXLL (that is not present in Delta or Gamma genera E6), is required to initiate degradation of E6AP. In either case, docking of an E6 protein at the E6AP LXXLL site was insufficient to initiate E6AP degradation.

## Discussion

HPV16 and BPV1 E6 both interact with extended LXXLL alpha-helical peptides, are stabilized by the association in vivo, and solubilized by the association in vitro, which led to their crystallization and structural determination [25, 41]. But significant questions remain. How do E6 proteins discriminate in interaction among many potential binding partners? Can we predict the interaction targets of different genera of E6 proteins? What are the additional structural features of E6 beyond interacting with a cellular target LXXLL docking site that determine the biological properties of E6? How are E6 proteins that associate with particular cellular proteins evolutionarily related to each other?

There are five genera of HPV. Our work demonstrates the Alpha genera is the sole genera where E6 targets E6AP while the remaining (Beta, Gamma, Nu, and Mu) preferentially target MAML1 or another protein(s) with a similar LXXLL motif as MAML1. While HPV41 E6 bound MAML1 preferentially, it was the only E6 protein that while preferentially binding MAML1 failed to repress MAML1 transactivation. It is possible that ancillary E6 structure(s) that may be important for transcriptional repression are not present in HPV41 E6, or that in the genera, E6 has different biological functions.

The crystal structures of BPV1 and HPV16 E6, together with sequence alignments of other E6 proteins predicted that other E6 proteins would also interact with acidic amphipathic alpha helical peptides [41]. The conservation of contact residues between BPV1 and HPV16 E6 and the LXXLL ligands, plus the recent discovery that MAML1 was a common target of E6 proteins from several genera, prompted us to speculate that the preferred LXXLL targets of diverse genera might be predictable from multiple sequence alignment data, and that MAML1 or MAML1-like targets would be the most common E6 binding targets of diverse genera of papillomaviruses. We have taken three separate approaches to test these hypotheses.

Examination of the crystal structures of BPV1 E6 plus the PXN LXXLL peptide, compared to HPV16 in complex with the LXXLL peptide of E6AP showed a distinctive difference to be hydrophobic BPV1 E6 contacts with M1 of the LXXLL PXN peptide compared to charged interactions between HPV16 E6 and glutamic acid at position -3 of the E6AP LXXLL peptide (Fig 2). To test the importance of this contact, we prospectively selected peptides from a large random peptide phage library using BPV1 E6 as a binding substrate, and found a strong selection for peptides that had a hydrophobic residue in the same position as M1 of the PXN or MAML1 peptides (Fig 1). This illustrates the importance of this hydrophobic interaction when peptides compete for interaction with BPV1 E6. In the comparison of HPV16 E6 and BPV1 E6, both experimental selection of peptides and examination of the structure highlighted the important interactions between E6 and position -3 of the LXXLL peptides. Because only two E6 structures have been determined so far; it is possible that differences between the E6AP and MAML1 peptides other than at position -3 are more important to other E6 proteins to discriminate between E6AP and MAML1 association.

In testing 45 E6 proteins for interaction with E6AP and MAML1, we were surprised that most E6 proteins had a clear binding preference, that few interacted with both, and then only at low levels, and that no E6 protein both stimulated E6AP degradation and repressed GAL4_MAML1 transcriptional activation (Fig 5). Why would an Alpha E6 protein not evolve to both interact with E6AP to target cellular proteins for degradation, and simultaneously also interact with MAML1 to repress the Notch tumor suppressor pathway in squamous epithelia (like a Beta or Gamma genus E6 protein)? Our experiments do not yet shed light on this question.

All E6 proteins that had a preference for association with E6AP also stimulated the degradation of E6AP, indicating the linkage of physical association with biological function. However, binding of an E6 protein to E6AP at the LXXLL site was not sufficient to initiate in vivo degradation of E6AP. This was demonstrated by the use of the E6AP_LDDLL chimera, where BPV1 E6 or Gamma genera HPV E6 (HPV types 4, 112 and 131) bound robustly to E6AP_Ub-_LDDLL yet failed to stimulate degradation of E6AP_LDDLL. In contrast, HPV16 E6 (which bound E6AP_Ub-_LDDLL relatively poorly) could still initiate degradation of E6AP_LDDLL, indicating the functionality of the E6AP_LDDLL mutant. Thus, while binding of an E6 protein to the LXXLL site of E6AP is necessary, it is not sufficient for degradation of E6AP (Fig 8). This implies that two properties of alpha genera E6, binding and initiation of degradation of E6AP, co-evolved as the Alpha and Alpha-clustered E6 proteins diverged from interaction with other LXXLL target proteins (possibly MAML1).

The Alpha-clustered genera we tested in this study, being from Alpha, DyoDelta, Dyopi, Omega, and Omikron genera, behaved similarly in binding to E6AP and stimulating its degradation. Phylogenetic clustering would suggest that Upsilon and Dyoomikron genera E6 proteins (that were not tested in this study) would also interact with E6AP and stimulate its degradation because they cluster together next to the Dyodelta, Omega, Omikron, and Alpha genera. These genera, where E6 proteins interact with E6AP and stimulate its degradation, are related in binding, in vivo biological effect, and phylogenetic clustering. We would propose that these genera should be clustered as a super-genera or clade in the description of papillomavirus oncoproteins. It is remarkable that it is not the presumed ultimate target of E6 action (such as p53, or type I cellular PDZ proteins [83] for the high-risk Alpha genera HPV E6) that phylogenetically clusters these diverse E6 proteins together, but is the common mechanism of MAML1 or E6AP association. While the activities of Alpha high-risk E6 proteins have been speculated to compensate for the activities of E7 (such as E7 activation of p53 leading to E6 mediated p53 degradation), within this larger super-genera, that is not the case, since the cetacean papillomaviruses (such as Omikron and Dyopipa genera) do not encode an E7 gene [5, 69], although additional E7-like functionality could be encoded elsewhere in the genome, such as within E6 or elsewhere. Earlier, it was customary in the field to think of papillomaviruses as either mucosal or cutaneous types. However within the Alpha genera, there are both cutaneous (like HPV types 7 and 10) and mucosal infections (like HPV types 11 and 16); similarly, both mucosal and cutaneous sites also harbor Beta and Gamma papillomaviruses [84, 85]. What is common to all Alpha compared to Beta or Gamma genera, is the preference of the E6 proteins for either E6AP or MAML1 association; this provides a simplified factor for categorizing many (but not all) papillomavirus types, compared to sequence divergence within L1 capsid sequence.

Our experiments suggest that E6 association with MAML1 is responsible for the clustering of most of the remaining E6 proteins that do not associate with E6AP. Only BPV1 E6 interacted with MAML3 in addition to MAML1, and only CPV7 E6 showed a preference for association of MAML2 compared to MAML1. To our surprise, rodent and canine MAML1 chimeras interacted with their cognate E6 proteins similarly to human MAML1, despite some amino acid differences in the LXXLL motif. This raises an unresolved question in how E6 association with MAML1 represses Notch signaling overall, since MAML1 can be deleted without ablating the gross development of a mouse. MAML1 -/- mice are born without gross developmental defects but are growth retarded and die at about 10 days (before weaning) and have lymphoid and muscle development defects [86, 87]. MAML3 null mice appear normal, but MAML3 combined with MAML1 deletion causes a severe defect in organogenesis similar to a Notch1 deficiency [88]. MAML2 null mice have not yet been described. Our results show that most E6 proteins interact with MAML1, with only BPV1 E6 interacting with both MAML1 and MAML3, and only CPV7 interacting preferentially with MAML2. MAML1, 2, and 3 are expressed in the skin at the RNA level by RNAseq analysis [89]. So how is it that E6 interaction with MAML1 represses Notch transcription overall when transcription complexes might be expected to alternatively contain MAML2 and/or MAML3 instead of MAML1? It is possible that individual MAML protein expression differs within the differentiating layers of squamous epithelium or that E6 may have a gain of function when bound to MAML1-Notch complexes to repress Notch signaling overall by an as yet uncharacterized mechanism, rather than acting upon MAML1 alone.

The E6 proteins that interacted poorly with either E6AP or MAML1, 2, or 3, and failed to either promote E6AP degradation or repress GAL4-MAML1 did not cluster with those that targeted either MAML1 or E6AP. OcPV1, similar to SfPV1 (also known as CRPV1 or Shope papillomavirus) has a different structure than most E6 proteins with three zinc structured domains, so it is not surprising that it does not share interaction targets with other canonical E6 proteins. The other non-E6AP/non-MAML1 interacting E6 proteins like TmPV1, TmPV2, RaPV1, and BpPV1 may have different LXXLL binding sites on cellular targets that remain to be determined. Alternatively, a technical reason such as incompatibility with the amino-terminal FLAG epitope tag may have prevented some of the tested E6 proteins from interacting well with either E6AP or MAML1.

Our results implicate E6 targeting of MAML1 in animal squamous cell carcinomas. Several of the MAML1-directed E6 proteins in this study were isolated from papillomaviruses in association with squamous cell cancers: OaPV3, CPV2, CPV3, CPV7, FcaPV2, and McPV2. OaPV3, CPV2, CPV3, and FcaPV2 all interact with MAML proteins, repress MAML1 transcriptional activation, and did not stimulate E6AP degradation. CPV7 and McPV2 did not bind MAML1 well by immunoprecipitation, but did repress GAL4_MAML1 transcriptional activation (Fig 7). Complete disruption of Notch signaling during development of the squamous epithelium of transgenic mice by either tissue specific Notch deletion [90, 91], epithelial deletion of the RPB/j -binding subunit of the Notch transcription complex [92, 93], or expression of a dominant negative MAML1 [94] all result in loss of squamous differentiation and squamous cell carcinomas. These results demonstrate that Notch signaling is a tumor suppressor in normal squamous epithelium, and the critical role of MAML1 in this process, making the targeting of MAML1 by these animal E6 proteins plausibly related to carcinogenesis. While these few papillomaviruses were associated with squamous cell cancers, most MAML1-targeting E6 proteins were not cancer-associated. This could reflect a low frequency of cancer development (similar to high-risk HPV types), the relative potency of different E6 proteins in repression of Notch, the possible differential sensitivity of developing compared to adult epithelia to Notch signaling disruption, and the role of E7 and or other oncoproteins in these animal papillomaviruses. Among the HPVs, Beta genera E6 proteins repress MAML1, but the consistent and continued expression of Beta or Gamma genus viral oncogenes in cutaneous squamous cell carcinomas has not been observed. A recent publication on Gamma genus HPV found the presence of HPV197 DNA associated with cutaneous squamous cell cancers, but the expression and role of viral early gene products in the cancers is as yet uncharacterized [3]. The role of Notch signaling in high-risk HPV infections and progression to cervical cancer has been contentious. Initially, Notch signaling was thought to be synergistically oncogenic with E6 and E7 in the development of cervical cancer, and experiments were presented demonstrating overexpression of Notch and synergistic transformation with papillomavirus oncogenes [95] which was then contradicted by different experimental approaches [96]. A study from the Kast lab [97] showed expression of high-risk E6 and E7 augmenting Notch1 expression while a recent study from the Doorbar lab [98] shows E6 repression of Notch1 expression through degradation of p53. While varying expression and cell culture systems have given rise to different results, head and neck cancers accumulate mutations in Notch signaling genes [99], and p53 null mice have normal appearing skin after development.

The phylogenetic alignment of E6 proteins shows a clear split between genera whose E6 proteins target E6AP and the genera that target MAML1. Consideration of the host species and the E6 sequence divergence give insight into when this occurred.

While some fish (SaPV1) and bird papillomaviruses express E6 proteins, these proteins are shorter and have only a single zinc structured domain, thus differing in structure from canonical E6 proteins with two zinc-structured domains that were tested here. It seems unlikely that fish and bird E6 proteins form a similar pocket for interaction with LXXLL motifs and as yet we have not tested these E6 proteins for either LXXLL interactions or modulation of Notch signaling. There is a single papillomavirus isolate from a marsupial (BpPV1) where the E6 protein has a canonical structure, but unfortunately our results with this E6 protein were ambiguous, binding both E6AP and MAML1 poorly, and neither significantly stimulating E6AP degradation nor repressing MAML1 transactivation (Fig 7). The Eutheria (placental mammals), diverged into two categories approximately 100M years ago: the Laurasiatheria (shrews, bats, ungulates, and cetaceans) and the Euarchontoglires (tree shrews, rodents, primates). Since E6 proteins that target E6AP have hosts within both of these groups of mammals, it is reasonable to speculate that the divergence of E6AP-directed E6 proteins must have predated the split between these two groups of Eutheria. Additional samples of papillomaviruses from marsupials, shrews, and rodents may provide further insight into the split between E6AP and MAML directed E6 proteins.

Among animal models of high-risk HPV infections, we show here that the Rhesus papillomavirus (Macaca mulatta Papillomavirus type 1, MmPV1, also known as RhPV1) E6 protein associates with E6AP, targets p53 for degradation (Fig 7 and S7 Fig), and causes genital squamous cell cancers, making this an excellent animal model of high-risk HPV disease [76]. Other animal papillomavirus infections do not model the activities of the Alpha genera high-risk HPV E6 and E7 proteins. Our findings suggest the possibility that additional sampling of rodents might reveal a papillomavirus whose E6 protein preferentially targets E6AP, which would apply the power of mouse genetic analysis to the activities of viral oncoproteins that are relevant to lethal human papillomavirus infections.

## Materials and methods

### Cells and tissue culture

E6AP-null 8B9 cells (A gift of Dr. Lawrence Banks, ICGEB, Italy) [100], and 293T cells (American Type Culture Collection) were maintained in DMEM media supplemented with 10% fetal bovine serum, glutamine and antibiotics. Transient transfections were performed using polyethylenimine (PEI). Transient Vaccinia virus expression of proteins was performed in CV-1 cells (American Type Culture Collection) as described [101].

### Plasmids

E6 genes were synthesized (Genewiz Corp.) using native unmodified codons, terminated at the native stop codon, and were cloned as amino-terminal 2X-FLAG-tagged fusions in pcDNA3. HA-tagged E6AP WT and E6AP containing a C843A mutation that eliminates ubiquitin ligase activity (E6AP_Ub-), HA-tagged MAML1, and GAL4-MAML1 fusions have been previously described [34]. cDNA’s for human MAML2 and MAML3 were generously provided by Brandon White (San Jose State University). Epitope-tagged E6AP, GFP, MAML, and E6 were all expressed from the pcDNA3 plasmid.

### Western blots

12-well plates of subconfluent HEK293T cells were transfected with 0.5 ug HA-tagged E6AP or E6AP-Ub-, 2 ug HA-MAML1, 1 ug FLAG-E6, and 0.02 ug HA-GFP (as an internal transfection control) with a total of 3.4 ug of DNA complexed with 4 ug of polyethylenemine (PEI). Eighteen hrs. later, cells were lysed on ice in 0.5 mls of 0.5X IGEPAL lysis buffer (1X IGEPAL lysis buffer contains contains 150 mM NaCl; 50 mM Tris pH 7.5; 0.5 mM Dithiothreitol (DTT); 50 mM NaF; 5 mM NaPPi; 1% IGEPAL; 0.01% phenylmethylsulfonyl flouride; 1mM sodium vanadate; 1ug/ml leupeptin/aprotinin), pelleted at 15,000 X G at 4°C for 15 min. The supernatant was immune precipitated with 10 ul anti-FLAG M2 antibody coupled beads (Sigma) for 1 hr. and then beads were washed three times with ice-cold 1.0 ml 0.5X lysis buffer. Samples were eluted with SDS and resolved on SDS-PAGE gels, blotted to PVDF membranes and probed sequentially with rabbit anti-HA antibody, and rabbit anti-FLAG antibodies (Sigma). Bound antibodies on the blot were detected with secondary antibodies coupled to horseradish peroxidase and chemiluminescent substrates, and images captured and analyzed using an Alpha-Innotech Fluorchem detector and associated image analysis software. For quantitation, input samples and immune precipitations were run in adjacent lanes for each transfection, and immune precipitations calculated as percent of input signal and then normalized to the input HA-GFP signal. Statistical analysis of normalized, independently replicated experiments was performed with Excel software. For E6AP degradation assays, 0.5 ug HA-E6AP, 1 ug FLAG-E6 proteins, 0.02 ug HA-GFP and 0.5 ug of empty pcDNA3 (total of 2 ug) were complexed with 3 ug PEI and transfected into HEK293T cells and harvested by denaturing lysis 18 hrs. post-transfection.

### Luciferase reporter assays

0.1 ug of GAL4_MAML1, 0.2 ug of FLAG_E6, 0.05 ng GAL4-responsive luciferase reporter, 0.01 ug pCMV renilla internal transfection control plus 0.64 ug pUC19 to a total of 1.0 ug DNA complexed with 3 ug PEI, was transfected into HEK293T cells and harvested 18 hrs. later. Duplicate wells for each sample were used in each assay and the assay was independently repeated 4 times for statistical analysis.

### Phage display

Glutathione S-Transferase (GST) or Chitin binding domain (CBD) were fused at the carboxy-terminus to a TEV protease cleavage site, a FLAG epitope and then BPV1 E6; the fusion protein was expressed overnight in CV-1 cells using the pTM1 vaccinia virus expression system [101]. Either Glutathione-agarose or Chitin-agarose beads were blocked overnight in 2% BSA in PBS with 0.3% IGEPAL. An aliquot of an M13 phage display library expressing random 12-mer peptides as fusions to the phage pIII gene (Ph.D.-12 library, New England Biolabs) containing 1.5 X 10^11^ phage was blocked overnight on ice in Luria Broth containing 1% BSA in PBS with 0.1% IGEPAL. A 6 cm plate of CV1 cells expressing the GST-BPV1 E6 or CBD-BPV1 E6 fusion proteins were lysed on ice in 250 ul 0.1 X IGEPAL lysis buffer containing 1% BSA. Cell lysates were clarified by centrifugation (15,000 XG for 30 min), and the supernatant applied to 10 ul of blocked glutathione-agarose or chitin-agarose beads rocking for 30 min. Beads were then washed three times with 1X Luria Broth containing 1% BSA, 0.1% IGEPAL, and 3 mM DTT, and phage applied in 100 ul Luria Broth containing 1% BSA, and 0.1% IGEPAL at 4°C for 1 hr. Beads were washed 6X with 1.0 ml of binding buffer, 1X with TEV protease cleavage buffer, transferred to a new tube and then cleaved with 5 U TEV protease in 25 ul of buffer (Invitrogen). Released phage were amplified and purified by PEG precipitation in XL1-blue bacteria according to the manufacturer’s instructions. Five rounds of phage display selection were performed starting with CBD as the fusion partner alternating with GST at each selection round. Individual phage were picked at the fifth round of selection and eluted, amplified, phage DNA isolated, and the pIII-peptide fusion segment DNA-sequenced.

### Phylogenetic analysis

Multiple protein sequence files were downloaded from the papillomavirus episteme [102], alignments were performed with the MUSCLE 3.8.31 program [103] using the phylogeny.fr toolset [104]. The output from MUSCLE was used as input for phylogenetic analysis and graphing using PhyML 3.1/3.0 aLRT [105], and tree rendering with TreeDyn 198.3 [106].

## Supporting information

S1 Fig Multiple sequence alignment of Alpha genera E6 proteins. A multiple sequence alignment was performed using the MUSCLE program [103], and the output then entered into Jalview2 [109] to generate the ClustalX colorized image. The four upward-pointing arrows at the bottom of the alignment indicate the positions of HPV16 E6 amino acids that interact with position -3 of the E6AP LXXLL motif as shown in Fig 1. The highlighted sequence in a dashed-red line box is HPV16 E6.
(TIF)

S2 Fig Multiple sequence alignment of Beta genera E6 proteins. A multiple sequence alignment was performed using the MUSCLE program [103], and the output then entered into Jalview2 [109] to generate the ClustalX colorized image. The four upward-pointing arrows at the bottom of the alignment indicate the relative positions of BPV1 E6 amino acids that interact with position -3 of the PXN LXXLL motif as shown in Fig 1. The highlighted sequence in dashed red box is HPV8 E6.
(TIF)

S3 Fig Multiple sequence alignment of Gamma genera E6 proteins. A multiple sequence alignment was performed using the MUSCLE program [103], and the output then entered into Jalview2 [109] to generate the ClustalX colorized image. The four upward-pointing arrows at the bottom of the alignment indicate the relative positions of BPV1 E6 amino acids that interact with position -3 of the PXN LXXLL motif as shown in Fig 1. The highlighted sequence in dashed red box is HPV4 E6.
(TIF)

S4 Fig Position of tested E6 proteins in a large set of animal and human papillomavirus E6 proteins. All E6 proteins sequences at papillomavirus episteme were downloaded and all HPV E6 were deleted except HPV types 1–50, 60, 112, 123, and 131 in order to decrease the overrepresentation of HPV sequences in the figure. Blue coloration identifies E6 proteins that primarily associate with MAML1 in this study, red primarily associate with E6AP. The bottom phylogenetic panel shows the distribution of tested E6 proteins in this study. MUSCLE [103] was used for the multiple sequence alignment, and the phylogram was generated using PhyML [105], and tree rendering with TreeDyn [106].
(TIF)

S5 Fig Preferential association of E6 proteins for either E6AP or MAML1. Parts A-E. HA-tagged E6AP_Ub-, MAML1 and GFP expression plasmids were co-transfected with the indicated FLAG-tagged E6 expression plasmids into 293T cells and harvested in 0.5X IPEGAL lysis buffer as described in the methods. Western blots of input samples are clustered at the top and FLAG immune precipitated samples at the bottom, except that lane 1 at the bottom cluster of blots is the same input sample from lane 1 in the top panel. Input was 4% of the immune precipitated sample size. E6* is a spliced E6 variant that does not co-immune precipitate with E6AP. Shown is a representative experiment out of three.
(TIF)

S6 Fig Targeted degradation of E6AP by overexpressed E6 proteins. Parts A-E. HA-tagged E6AP, and GFP expression plasmids were co-transfected with the indicated FLAG-tagged E6 expression plasmids into 293T cells and harvested SDS sample buffer 18 hrs. post transfection. E6* is a spliced E6 variant. Shown is a representative experiment out of three.
(TIF)

S7 Fig Alpha genera MmPV1 E6 degrades p53_hum in an E6AP-dependent manner. A. 8B9 E6AP-null mouse kidney epithelial cells [100] were co-transfected with 2X FLAG-E6AP, human p53 (hum_p53), and the indicated E6 proteins and lysed 18 hours post-transfection in 0.5X IGEPAL lysis buffer. p53 expression levels were determined using western blot. Both high-risk HPV16 E6 and MmPV1 E6 degrade p53 in an E6AP-dependent manner. Low-risk HPV11 E6 does not target p53 for degradation with or without co-expressed E6AP. B. Quantified p53 levels normalized to HA-GFP and p53 levels in the absence of E6 protein.
(TIF)

S8 Fig Canine and murine papillomavirus E6 proteins interact similarly with human, human-dog or human-mouse chimeric MAML1 proteins. A. The indicated FLAG tagged E6 proteins were co-transfected with MYC tagged human MAML1 and HA-tagged human-dog chimeric MAML1 where the last 12 amino acids of human MAML1 sequence encompassing the LXXLL E6 binding site was replaced with the canine LXXLL sequence. 293T cells were transfected, lysed after 18 hrs and immune precipitated using FLAG antibody beads. The blot was sequentially probed with rabbit anti-HA, mouse anti-MYC clone 9B11, and then rabbit anti-FLAG. B. Performed as in part A but with HA-tagged human-murine MAML1 where the the last 12 amino acids of human MAML1 sequence encompassing the LXXLL E6 binding site was replaced with the murine LXXLL sequence.
(TIF)

S9 Fig Clustering of E6 function among a large set of animal and human papillomavirus E6 proteins. All E6 protein sequences at papillomavirus episteme were downloaded and all HPV E6 were deleted except HPV types 1–50, 60, 112, 123, and 131 in order to decrease the overrepresentation of HPV sequences in the figure. Blue coloration identifies E6 proteins that physically and functionally associate with MAML1 in this study, red those that physically and functionally associate with E6AP, and black for tested E6 proteins that were neither E6AP nor MAML1 directed. The small skull indicates samples in the tested set that were isolated in association with squamous cell cancers. MUSCLE [103] was used for the multiple sequence alignment, and the phylogram was generated using PhyML [105], and tree rendering with TreeDyn [106].
(TIF)

S1 Table MAML and E6AP LXXLL motifs by host species. LXXLL motifs from E6AP (UBE3A), MAML1, MAML2, and MAML3 are shown for each of the papillomavirus host species tested in the papillomavirus study set. NA indicates that a host species for the indicated gene was not found in Genbank. Underlinded amino acids in the LXXLL motifs indicate differences from the human LXXLL motifs.
(DOCX)
