# Supplementary figures and images for: Association of papillomavirus E6 proteins with either MAML1 or E6AP clusters E6 proteins by structure, function, and evolutionary relatedness
Source: PLoS Pathog. 2017 Dec 27;13(12):e1006781. doi: 10.1371/journal.ppat.1006781 (PMC5760104; doi:10.1371/journal.ppat.1006781)

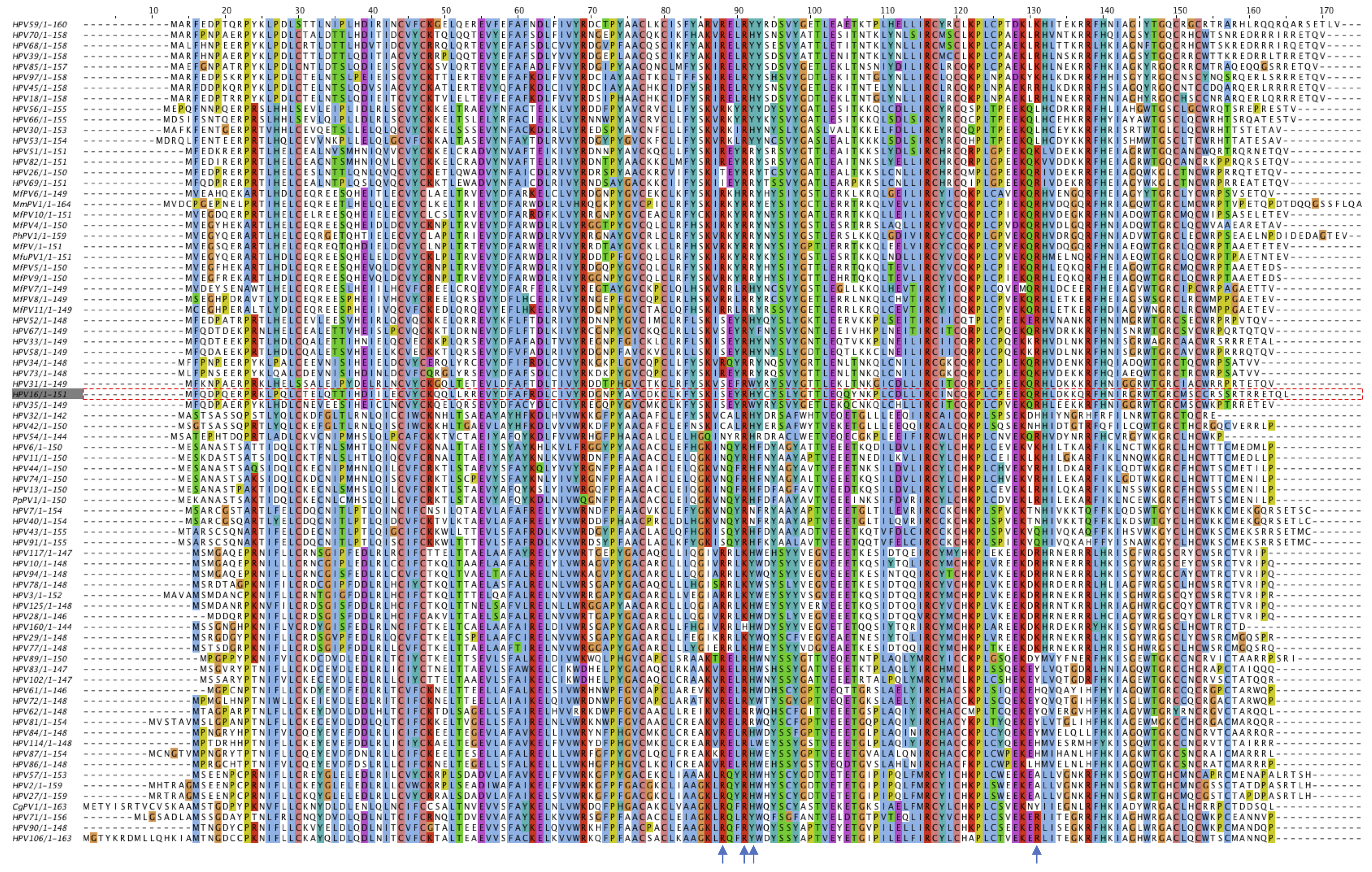

Supplement: S1 Fig — A multiple sequence alignment was performed using the MUSCLE program [103], and the output then entered into Jalview2 [109] to generate the ClustalX colorized image. The four upward-pointing arrows at the bottom of the alignment indicate the positions of HPV16 E6 amino acids that interact with position -3 of the E6AP LXXLL motif as shown in Fig 1. The highlighted sequence in a dashed-red line box is HPV16 E6. (TIF) [file ppat.1006781.s001.tif]

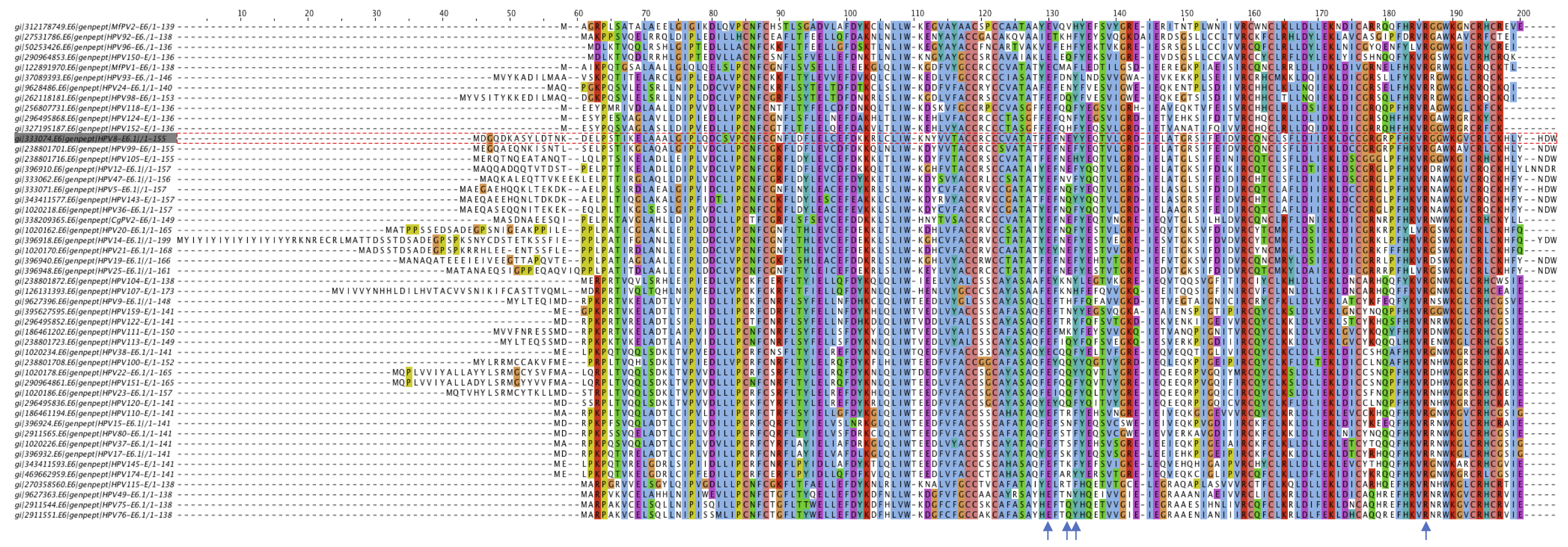

Supplement: S2 Fig — A multiple sequence alignment was performed using the MUSCLE program [103], and the output then entered into Jalview2 [109] to generate the ClustalX colorized image. The four upward-pointing arrows at the bottom of the alignment indicate the relative positions of BPV1 E6 amino acids that interact with position -3 of the PXN LXXLL motif as shown in Fig 1. The highlighted sequence in dashed red box is HPV8 E6. (TIF) [file ppat.1006781.s002.tif]

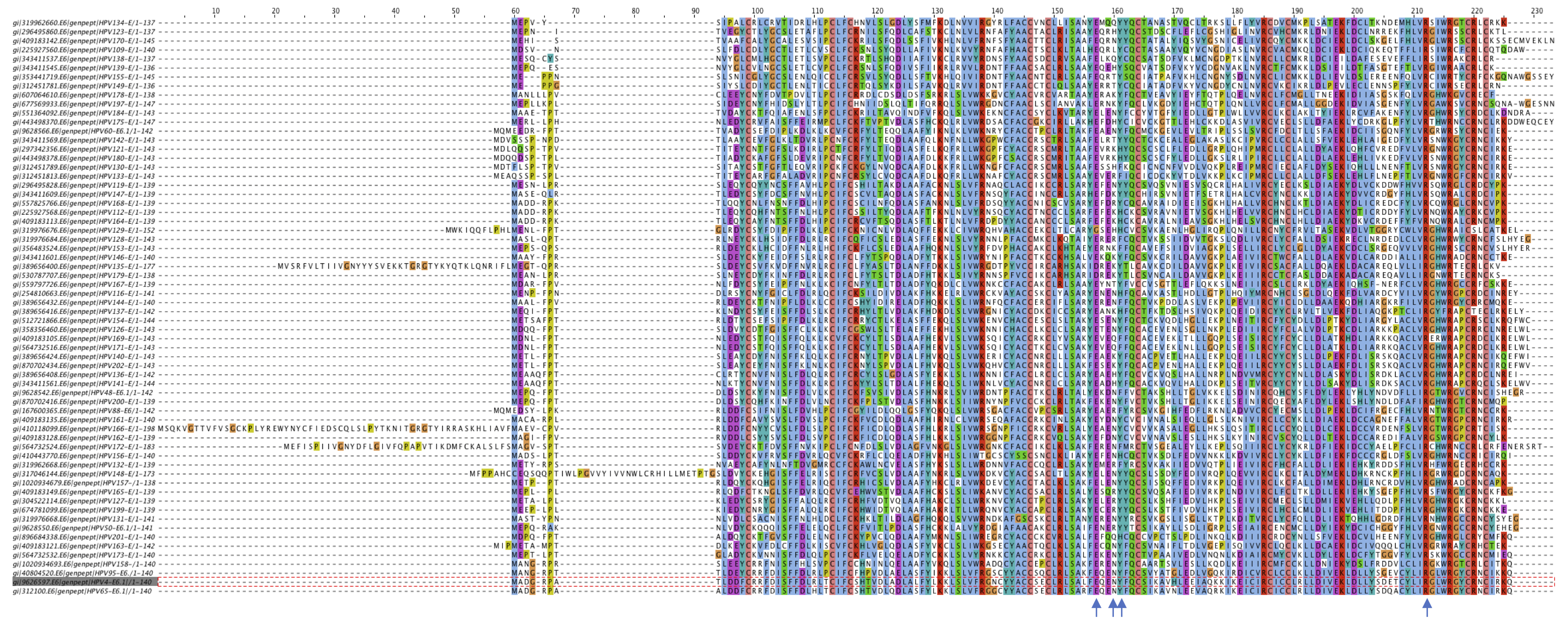

Supplement: S3 Fig — A multiple sequence alignment was performed using the MUSCLE program [103], and the output then entered into Jalview2 [109] to generate the ClustalX colorized image. The four upward-pointing arrows at the bottom of the alignment indicate the relative positions of BPV1 E6 amino acids that interact with position -3 of the PXN LXXLL motif as shown in Fig 1. The highlighted sequence in dashed red box is HPV4 E6. (TIF) [file ppat.1006781.s003.tif]

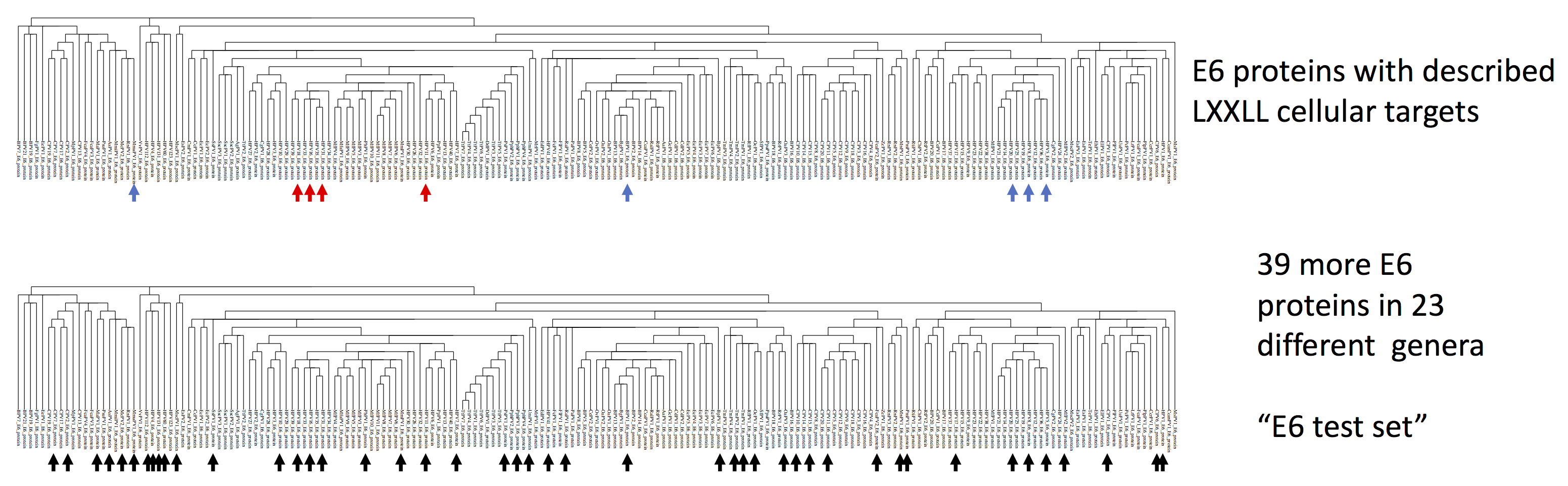

Supplement: S4 Fig — All E6 proteins sequences at papillomavirus episteme were downloaded and all HPV E6 were deleted except HPV types 1–50, 60, 112, 123, and 131 in order to decrease the overrepresentation of HPV sequences in the figure. Blue coloration identifies E6 proteins that primarily associate with MAML1 in this study, red primarily associate with E6AP. The bottom phylogenetic panel shows the distribution of tested E6 proteins in this study. MUSCLE [103] was used for the multiple sequence alignment, and the phylogram was generated using PhyML [105], and tree rendering with TreeDyn [106]. (TIF) [file ppat.1006781.s004.tif]

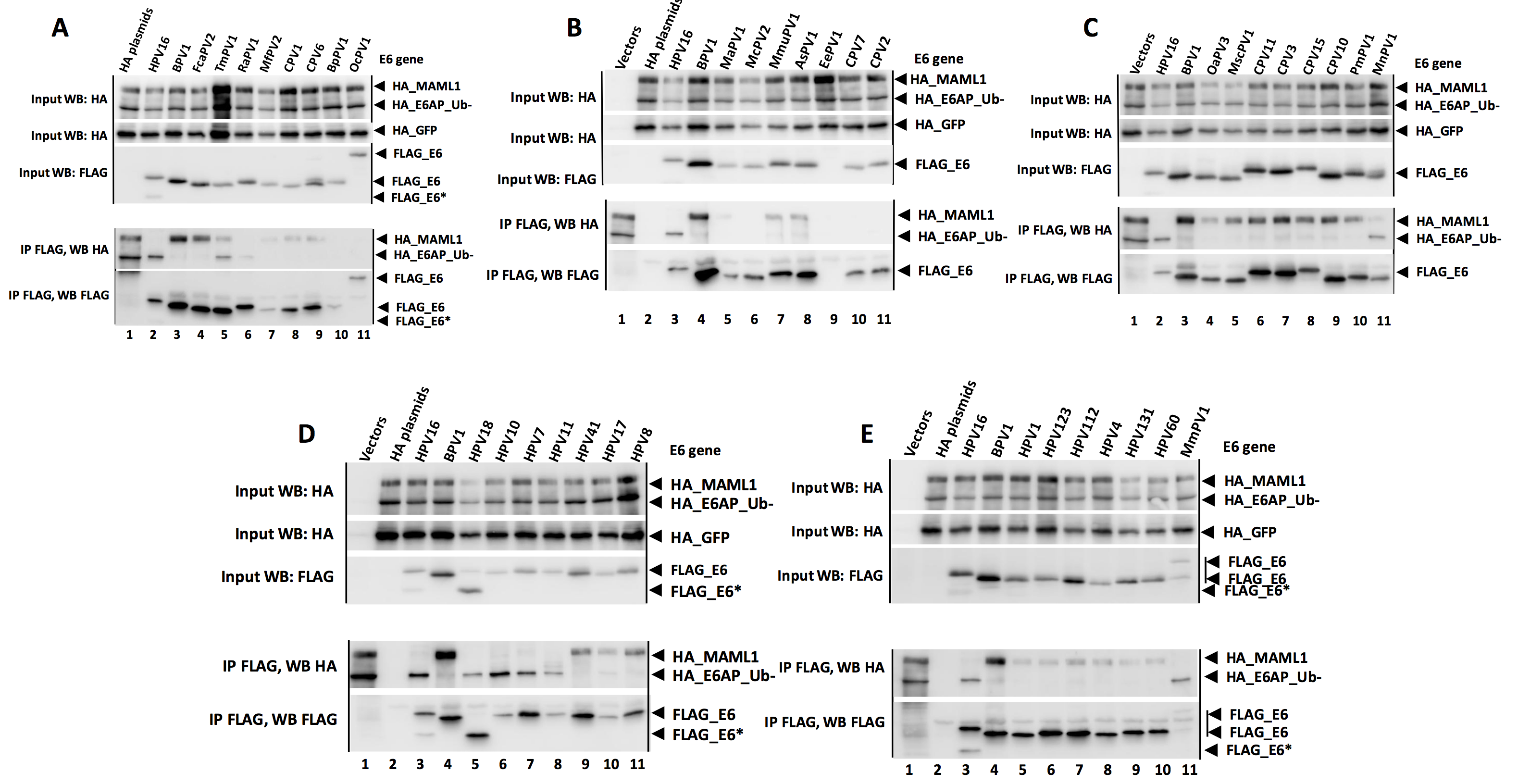

Supplement: S5 Fig — Parts A-E. HA-tagged E6AP_Ub-, MAML1 and GFP expression plasmids were co-transfected with the indicated FLAG-tagged E6 expression plasmids into 293T cells and harvested in 0.5X IPEGAL lysis buffer as described in the methods. Western blots of input samples are clustered at the top and FLAG immune precipitated samples at the bottom, except that lane 1 at the bottom cluster of blots is the same input sample from lane 1 in the top panel. Input was 4% of the immune precipitated sample size. E6* is a spliced E6 variant that does not co-immune precipitate with E6AP. Shown is a representative experiment out of three. (TIF) [file ppat.1006781.s005.tif]

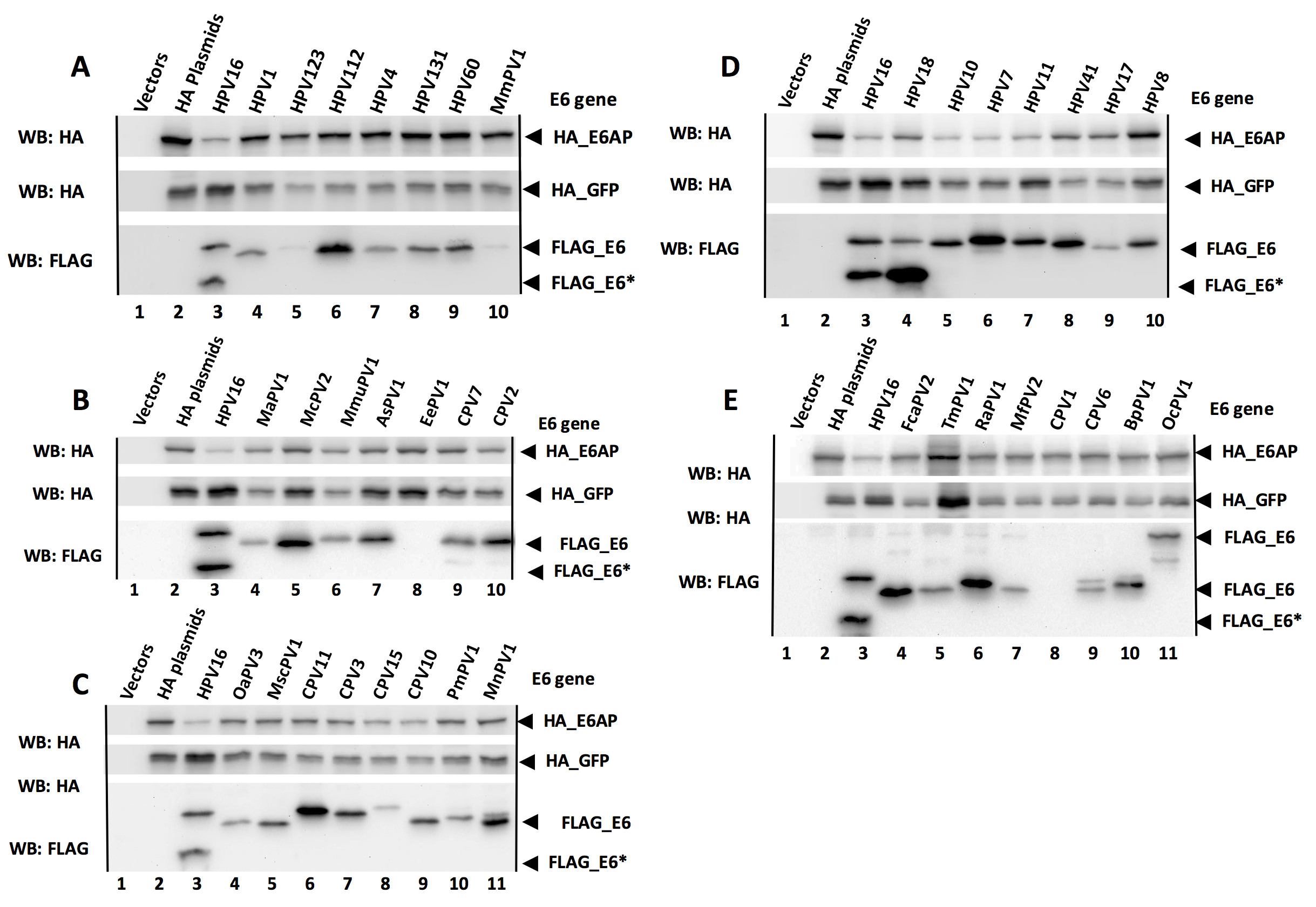

Supplement: S6 Fig — Parts A-E. HA-tagged E6AP, and GFP expression plasmids were co-transfected with the indicated FLAG-tagged E6 expression plasmids into 293T cells and harvested SDS sample buffer 18 hrs. post transfection. E6* is a spliced E6 variant. Shown is a representative experiment out of three. (TIF) [file ppat.1006781.s006.tif]

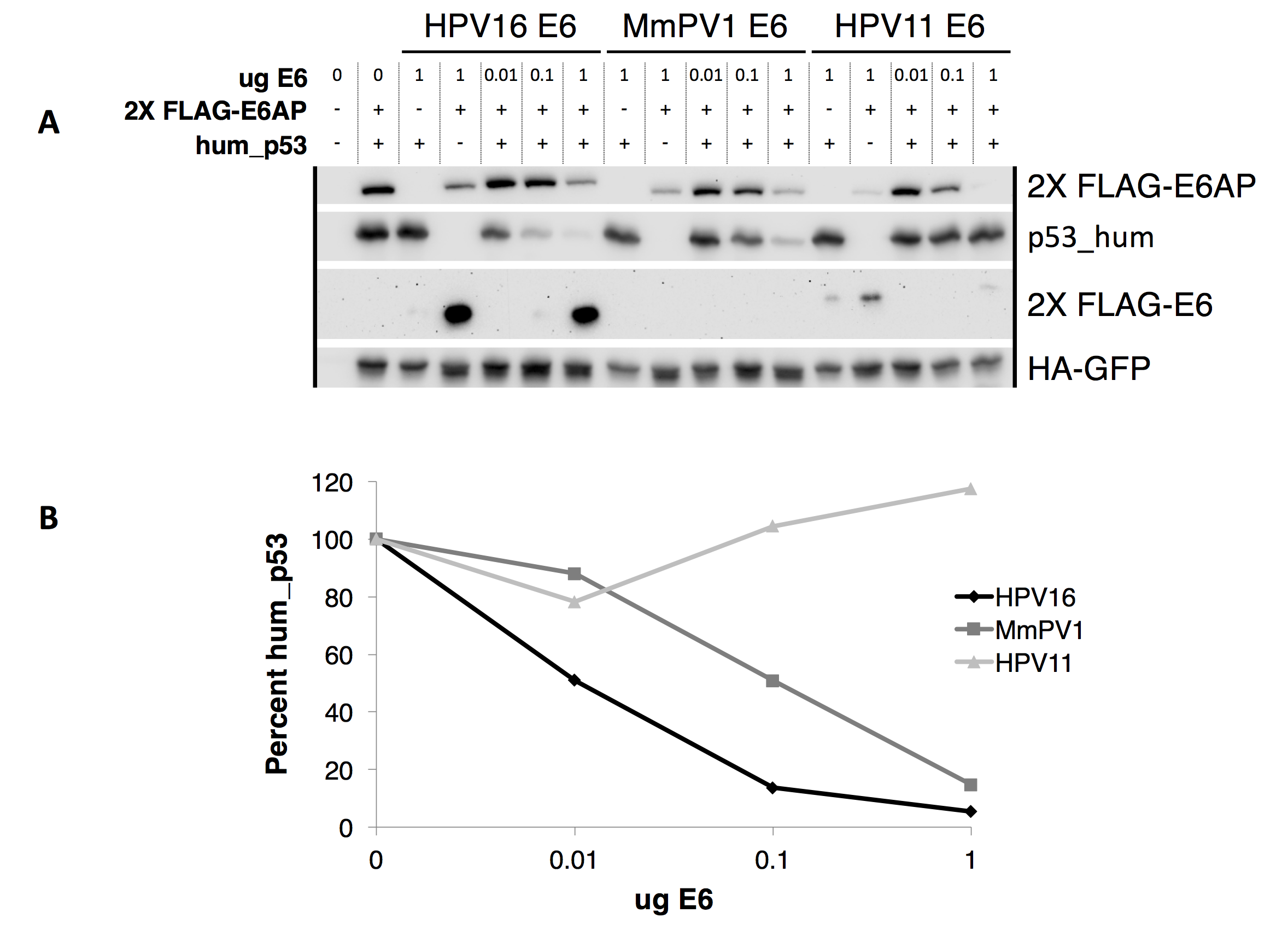

Supplement: S7 Fig — A. 8B9 E6AP-null mouse kidney epithelial cells [100] were co-transfected with 2X FLAG-E6AP, human p53 (hum_p53), and the indicated E6 proteins and lysed 18 hours post-transfection in 0.5X IGEPAL lysis buffer. p53 expression levels were determined using western blot. Both high-risk HPV16 E6 and MmPV1 E6 degrade p53 in an E6AP-dependent manner. Low-risk HPV11 E6 does not target p53 for degradation with or without co-expressed E6AP. B. Quantified p53 levels normalized to HA-GFP and p53 levels in the absence of E6 protein. (TIF) [file ppat.1006781.s007.tif]

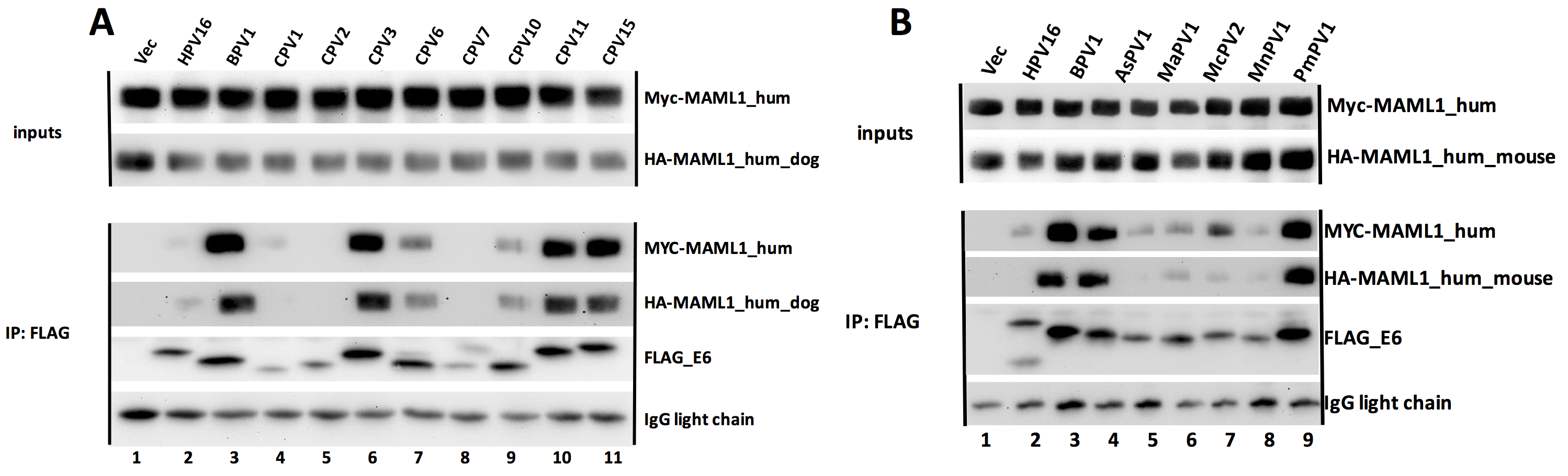

Supplement: S8 Fig — A. The indicated FLAG tagged E6 proteins were co-transfected with MYC tagged human MAML1 and HA-tagged human-dog chimeric MAML1 where the last 12 amino acids of human MAML1 sequence encompassing the LXXLL E6 binding site was replaced with the canine LXXLL sequence. 293T cells were transfected, lysed after 18 hrs and immune precipitated using FLAG antibody beads. The blot was sequentially probed with rabbit anti-HA, mouse anti-MYC clone 9B11, and then rabbit anti-FLAG. B. Performed as in part A but with HA-tagged human-murine MAML1 where the the last 12 amino acids of human MAML1 sequence encompassing the LXXLL E6 binding site was replaced with the murine LXXLL sequence. (TIF) [file ppat.1006781.s008.tif]

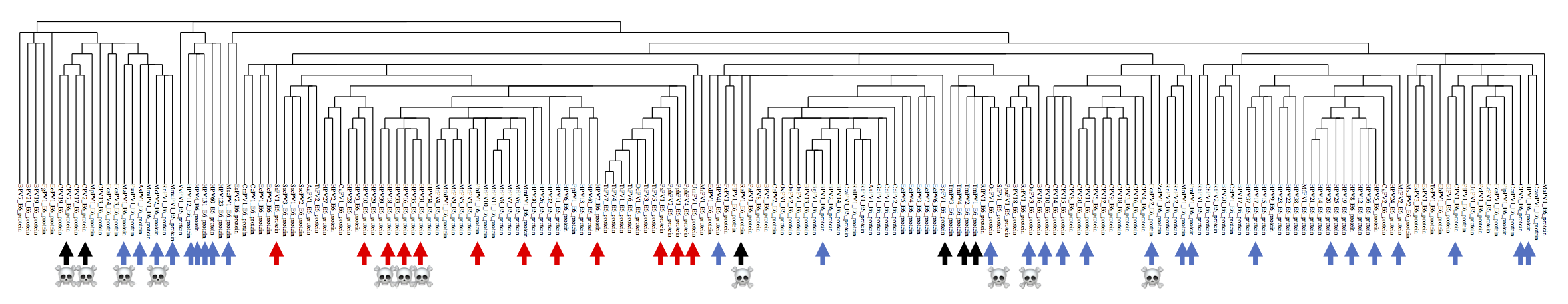

Supplement: S9 Fig — All E6 protein sequences at papillomavirus episteme were downloaded and all HPV E6 were deleted except HPV types 1–50, 60, 112, 123, and 131 in order to decrease the overrepresentation of HPV sequences in the figure. Blue coloration identifies E6 proteins that physically and functionally associate with MAML1 in this study, red those that physically and functionally associate with E6AP, and black for tested E6 proteins that were neither E6AP nor MAML1 directed. The small skull indicates samples in the tested set that were isolated in association with squamous cell cancers. MUSCLE [103] was used for the multiple sequence alignment, and the phylogram was generated using PhyML [105], and tree rendering with TreeDyn [106]. (TIF) [file ppat.1006781.s009.tif]
