# Supplementary material for: Association of papillomavirus E6 proteins with either MAML1 or E6AP clusters E6 proteins by structure, function, and evolutionary relatedness
Source: PLoS Pathog. 2017 Dec 27;13(12):e1006781. doi: 10.1371/journal.ppat.1006781 (PMC5760104; doi:10.1371/journal.ppat.1006781)
Supplement: S1 Table — LXXLL motifs from E6AP (UBE3A), MAML1, MAML2, and MAML3 are shown for each of the papillomavirus host species tested in the papillomavirus study set. NA indicates that a host species for the indicated gene was not found in Genbank. Underlinded amino acids in the LXXLL motifs indicate differences from the human LXXLL motifs. (DOCX) [file ppat.1006781.s010.docx]

| **Species name** | **Common name** | **MAML1** | **MAML2** | **MAML3** | **E6AP** |
| --- | --- | --- | --- | --- | --- |
| *Homo Sapiens* | human | wmsdlddllgsq | kdinldeilgnns | wmqeldelfgnp | eltlqellgee |
| *Canis lupus familiaris* | dog | wmndlddllgpq | kdinldeilgnns | wmqeldelfgnp | eltlqellgee |
| *Felis catus* | cat | wmndlddllgpq | kdinldeilgnns | wmqeldelfgnp | eltlqellgee |
| *Rattus norvegius* | rat | winelddllgsq | kdinldeilgsns | wmqeldelfgnp | eltlqellgee |
| *Mus musculus* | house mouse | winelddllgsq | kdinldeilgsns | wmqeldelfgnp | eltlqellgde |
| *Apodemus sylvaticus* | wood mouse | na^1^ | na | na | na |
| *Peromyscus maniculatus* | prairie deer mouse | winelddllgsq | kdinldeilgsns | wmqeldelfnp | eltlqellgee |
| *Mesocricetus auratus* | golden hamster | winelddllgsq | kdinldeilgsns | wmqeldelfgnp | eltlqellgee |
| *Phodopus sungorus* | siberian hamster | na | na | na | na |
| *Oryctolagus cuniculus* | european rabbit | na | kdinldeilgnnss | wmqeldelfgnp | eltlqellgee |
| Sylvilagus floridanus | eastern cottontail rabbit | na | na | na | na |
| *Bettongia penicillata* | woylie | na | na | na | na |
| *Cervus* | elk | na | na | na | eltlqellgee |
| *Miniopterus natalensis* | long-fingered bat | wmndlddllgpq | kdinldeilgnnn | na | eltlqellgee |
| *Rousettus aegyptiacus* | fruit bat | wmsdlddllgpq | kdinldeilgnns | wmqeldelfgnp | eltlqellgee |
| *Miniopterus schreibersii* | bentwing bat | na | na | na | na |
| Bos taurus | domestic cattle | wmsdlddllgpq | kdinldeilgnns | wmqeldelfgnp | eltlqellgee |
| *Sus scrofa domesticus* | domestic pig | wmndlddllgpq | na | wmqeldelfgnp | eltlqellgee |
| *Tursiops truncates* | bottlenose dolphin | wmndlddllgpq | kdinldeilgnns | wmqeldelfgnp | eltlqellgee |
| *Phocoena phocoena* | harbour porpoise | na | na | na |  |
| *Trichechus manatus* | manatee | windlddllgsq | kdinldeilgnns | wmqeldelfgnp | eltlqellgee |
| *Ursus maritimus* | polar bear | wmndlddllgpq | wmkdinldeilgnns | na | eltlqellgee |
| *Ovis aries* | sheep | wmsdlddllgpq | kdinldeilgnns | wmqeldelfgnp | eltlqellgee |
| *Danio rerio* | zebra fish | wmddidellashq | na | wmeeffpnq | eltlqellgee |

The sequences of translated LXXLL motifs from mRNA sequences of MAML1, 2, and 3 and E6AP for each of the host species of the test set of E6 proteins. Red denotes changes in sequence from *Homo sapiens*.

^1^na—not available in Genbank
